# Implanting Ni-O-VOx sites into Cu-doped Ni for low-overpotential alkaline hydrogen evolution

**DOI:** 10.1038/s41467-020-16554-5

**Published:** 2020-06-01

**Authors:** Yibing Li, Xin Tan, Rosalie K. Hocking, Xin Bo, Hangjuan Ren, Bernt Johannessen, Sean C. Smith, Chuan Zhao

**Affiliations:** 1 School of Chemistry, The University of New South Wales, Sydney, NSW 2052 Australia; 2 Integrated Materials Design Laboratory, Department of Applied Mathematics, Research School of Physics, The Australian National University, Canberra, ATC 2601 Australia; 3 Department of Chemistry and Biotechnology, Centre for Translational Atomaterials and ARC Training Centre for Surface Engineering for Advanced Material SEAM, Swinburne University of Technology, Hawthorn, VIC 3122 Australia; 4 ANSTO Australian Synchrotron, 800 Blackburn Rd, Clayton, VIC 3168 Australia

**Keywords:** Electrocatalysis, Hydrogen fuel, Electrocatalysis

## Abstract

Nickel-based catalysts are most commonly used in industrial alkaline water electrolysis. However, it remains a great challenge to address the sluggish reaction kinetics and severe deactivation problems of hydrogen evolution reaction (HER). Here, we show a Cu-doped Ni catalyst implanted with Ni-O-VOx sites (Ni(Cu)VOx) for alkaline HER. The optimal Ni(Cu)VOx electrode exhibits a near-zero onset overpotential and low overpotential of 21 mV to deliver –10 mA cm^−2^, which is comparable to benchmark Pt/C catalyst. Evidence for the formation of Ni-O-VOx sites in Ni(Cu)VOx is established by systematic X-ray absorption spectroscopy studies. The VOx can cause a substantial dampening of Ni lattice and create an enlarged electrochemically active surface area. First-principles calculations support that the Ni-O-VOx sites are superactive and can promote the charge redistribution from Ni to VOx, which greatly weakens the H-adsorption and H_2_ release free energy over Ni. This endows the Ni(Cu)VOx electrode high HER activity and long-term durability.

## Abstract (web summary)

Producing H_2_ from water using electricity and earth-abundant elements is necessary for worldwide renewable fuel production, yet most electrocatalysts have sluggish activities or poor stabilities. Here, authors show vanadium oxide modified copper-doped nickel to enable active and durable H_2_ evolution.

## Introduction

The development of cost-effective, highly active, and stable catalysts is a critical element for societal pursuit of sustainable energy. Hydrogen evolution reaction (HER) via water electrolysis is one of the simplest and efficient methods for H_2_ production. Currently, Pt-based precious metals have been considered to be the most effective electrocatalysts for HER^1,2^, but the high cost and scarcity of noble metals greatly hamper their practical utilization. To develop catalysts capable of sustainable H_2_ generation, tremendous efforts have been made to develop earth-abundant materials to replace precious-metal-based catalysts^3–10^. Particularly, Ni and stainless steel are used as cathode materials for industrial water electrolysis, which exhibit relatively high electrocatalytic activity toward HER in alkaline solution^11,12^. However, these materials often experience significant deactivation during prolonged water electrolysis because of strong hydrogen adsorption and/or chemical corrosion by oxygen diffusion on Ni^13–15^.

Currently, few first-row transition-metal-based catalysts can compete with Pt/C catalyst in alkaline media where protons are insufficient. Therefore, the importance of developing HER catalysts that are highly efficient and stable in alkaline electrolyte has always been emphasized. For example, it is reported that coating NiO/Ni with Cr_2_O_3_ can promote high activity and stability towards HER^16^. Performance study revealed that Cr_2_O_3_ coating was essential for maintaining the core NiO/Ni active sites from oxidation and aggregation. Recently, we demonstrated that N-modified Ni catalysts with appropriate N coverage level plays a critical role towards electrochemical water splitting^17^. Experimental and density functional theory (DFT) calculations revealed that an appropriate N-coverage level can lead to favorable kinetics for H* adsorption and fast release of H_2_, and consequently enhanced activity and stability. Although precious-metal-free catalysts with high activity in alkaline electrolyte have been reported^4,18,19^, highly durable electrocatalysts that can be fabricated by a facile synthetic approach is highly desired to meet the requirements of commercial electrolyzers.

Vanadium oxides represent a family of compounds (such as V_2_O_3_, VO_2_, and V_2_O_5_) that have been exploited in a wide range of applications including energy storage^20^, catalysis^21^, and gasochromic coloration^22^. In industrial water electrolysis, V_2_O_5_ is often used as an additive to the alkaline electrolyte. It was found that V_2_O_5_ in the electrolyte can initiate reactivation of the deactivated Ni cathode, by formation of a vanadium-rich deposit that enables efficient hydride removal from Ni to refresh the active sites^23^. Inspired by this finding, in this study, we set out to implant highly active Ni–O–VOx sites into Cu-doped Ni to construct an efficient and stable HER catalyst by using a facile electrodeposition method. The Ni(Cu)VOx electrode exhibits a near-zero onset overpotential, high activity, and stability towards HER, ranking it one of the most efficient non-noble-metal HER catalysts reported to date. X-ray absorption spectroscopy (XAS) and DFT calculations verify the existence of Ni–O–VOx sites, which not only promote the formation of highly disordered metallic Ni structures, but induce electron transfer from Ni to VOx. This facilitates H-adsorption and fast H_2_ release, resulting in the excellent HER activity and stability.

## Results

### Synthesis and characterization of the Ni(Cu)VOx catalyst

**Fig. 1:**
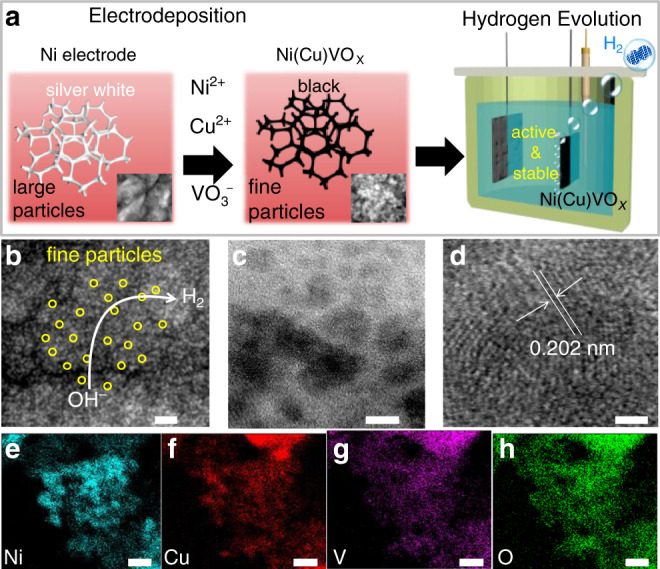
Sample preparation and morphology of the Ni(Cu)VOx electrode. **a** Schematic to prepare the Ni(Cu)VOx electrode for HER electrolysis. **b** High resolution SEM (scale bar: 100 nm). **c**, **d** HR-TEM (scale bar: 5 nm for **c** and 1 nm for **d**), and **e**–**h** TEM-EDS mapping images (scale bar: 25 nm).

The Ni(Cu)VOx catalyst was fabricated by a facile electrodeposition process on nickel foam (NF) unless otherwise stated (Fig. 1a, see details in “Methods” section and Supplementary Methods). In a typical synthesis, the electrodeposition was conducted in an electrolyte containing 0.5 M NiSO_4_, 12.5 mM CuSO_4_, 7 mM NH_4_VO_3_ and 0.5 M H_3_BO_3_ by dissolving each chemical in 50 mL Milli-Q water under sonication and then deposited at –2.0 V (vs. saturated calomel electrode (SCE)) for 600 s at room temperature. For physical characterization purpose, fluorine-doped tin oxide (FTO) glass was used as the substrate to eliminate the influence of Ni signal from NF substrate. Cyclic voltammetry (CV) was applied to study the electrochemical behavior of NH_4_VO_3_ and to deposit pure vanadium oxide cluster (VOx) on FTO. Shown in Supplementary Fig. 1, the NH_4_VO_3_ reduction peak appears at –2.09 V (vs. SCE) and the reverse scan produces a current loop at –2.02 and –1.76 V, indicative of a nucleation and growth mechanism for the deposition process^24,25^. The black-colored FTO also confirms the successful deposition of VOx. Ni(Cu)VOx and other control samples exhibit black color, except for Ni (Supplementary Fig. 2). The electrodeposition behaviors of Ni^2+^, Cu^2+^, and VO^3−^ were also characterized on NF. As shown in Supplementary Fig. 3, at –2.0 V (vs. SCE), the metal precursors all can be electrodeposited simultaneously onto NF. The different deposition potentials of each metal can influence the electrodeposition process and subsequently the composition of the film. However, in our case, the Ni^2+^ concentration (0.5 M) is 40 times higher than Cu^2+^ and 71.4 times higher than VO^3−^, therefore Ni deposition dominate, especially at the very negative potentials e.g., –2.0 V. This is evident that at negative potentials (< –1.25 V), the deposition curve is very close to that of Ni, indicating Ni is the dominante element in the electrodeposited film, whilst Cu and VOx are doped into the structure of Ni.

The crystal structures of the prepared catalysts were characterized by X-ray diffraction (XRD). As shown in Supplementary Fig. 4, the XRD patterns for Ni and Ni(Cu) are all crystalline with three main diffraction peaks at 2*θ* = 44.44°, 51.26°, and 76.35°, assigning to (111), (200), and (220) planes of face-centered cubic (*fcc*) Ni (JCPDS, No.70-0989)^17,26^. For Ni(Cu), the shoulder peaks appear at the base of Ni(111) and (200), indicating the partial formation of NiCu alloy. There is a profound impact of VOx on the crystallization degree of Ni. The characteristic Ni peaks vanish in the VOx-modified Ni sample (NiVOx), suggesting its highly disordered structure. For Ni(Cu)VOx, a small and broad peak at 44.44° was observed, which suggests the poor crystallization and the formation of fine metallic Ni nanoparticles (NPs), as well as that a portion of Ni crystalline structures were restored with the incorporation of Cu. The size of Ni(Cu)VOx NPs was calculated to be 4.6 nm according to Scherrer equation, which is much smaller than that of 26.8 nm for Ni and 19.1 nm for Ni(Cu). Furthermore, to see if the NH_4_VO_3_ precursor was reduced to metallic V or not, we obtained the XRD spectra of the electrodeposited pure VOx. As shown in Supplementary Fig. 5 and Supplementary Note 1, the electrodeposited VOx is amorphous and there are no new peaks that can be assigned to metallic V. This result is in consistent with the X-ray photoelectron spectroscopy (XPS) and XAS observations, where only oxidized V is observed (see below).

The VOx-induced formation of fine Ni NPs was further verified by morphology studies. Scanning electron microscopy (SEM) images show fine NPs with a size around 5 nm were obtained for Ni(Cu)VOx (Fig. 1b and Supplementary Fig. 6), while much larger crystals were observed for Ni and Ni(Cu), further suggesting the deposited VOx play an important role to grain refinement. The transmission electron microscopy (TEM) image also shows fine Ni(Cu)VOx NPs having the size of 4–5 nm (Fig. 1c). It is noteworthy that discernible and undecipherable lattice fringe coexist, meaning these NPs are poorly crystallized. A closer investigation of the HRTEM result shows *d*-spacing of 0.202 nm (Fig. 1d), corresponding to (111) plane of *fcc* Ni. The typical TEM-EDS mapping shows the uniform distribution of Ni, Cu, V, and O (Fig. 1e–h).

The elemental composition of the Ni(Cu)VOx catalyst was studied by XPS (Supplementary Fig. 7). For Ni2p, the binding energy at 852.8 eV (Ni2p3/2 line) and 870.0 eV (Ni2p1/2 line) with peak split spin–orbit components of 17.2 eV, illustrate the formation of metallic Ni^27^. In addition, the peak at ~855.9 eV is attributed to Ni^2+^, indicating Ni is partially oxidized as a result of deposited VOx (see XAS data below). The Cu2p shows the Cu2p3/2 at 932.8 eV and Cu2p1/2 at 952.6 eV, respectively, with a Cu2p peak split spin-orbit components of 19.8 eV, indicating copper is in metallic state. The binding energies for V2p3/2 show two peaks at 517.1 eV (V^5+^) and 516.2 eV (V^4+^), suggesting the formation of VOx with lower vanadium oxidation states^28^. The fitting peak of O1s shows three peaks at 529.7 eV corresponding to metal-O bond, 531.2 eV corresponding to vanadium oxide deposits, and 532.9 eV corresponding to oxygen species in the surface-adsorbed H_2_O molecule. The XPS spectra for Ni(Cu) and NiVOx were carried out as well. For Ni(Cu), the Ni2p and Cu2p XPS spectra show both the metallic Ni and Cu and slightly oxidized Ni and Cu, which are similar to Ni(Cu)VOx. For NiVOx, a higher oxidation degree of Ni was observed (Supplementary Fig. 8).

### Evaluation of electrocatalytic activity and stability

**Fig. 2:**
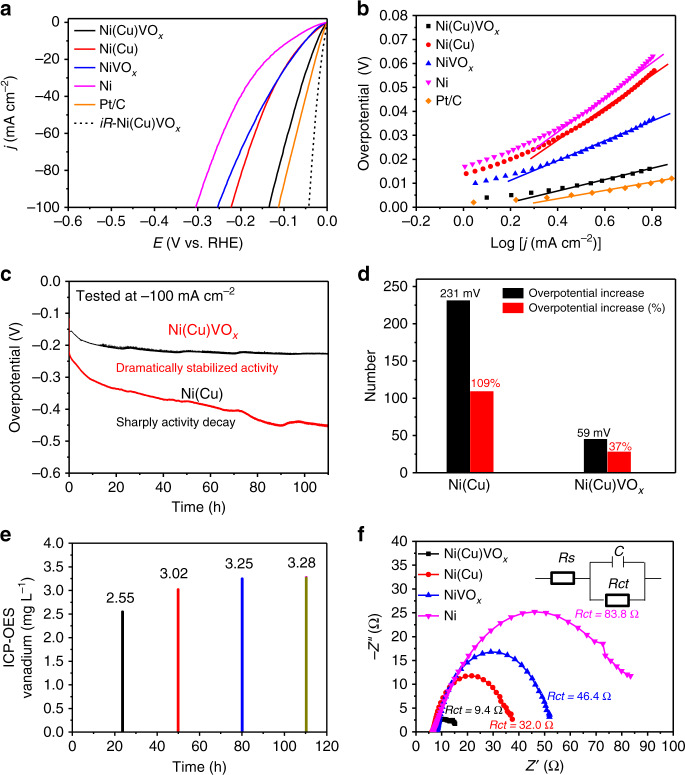
Electrochemical characterization of the Ni(Cu)VOx electrode. **a** LSV curves and **b** Tafel plots for Ni(Cu)VOx and control samples without *iR* compensation. The black dotted curve is the HER activity on Ni(Cu)VOx electrode with *iR* correction. **c** Chronopotentiometric curve of Ni(Cu)VOx in comparison with Ni(Cu). **d** Catalytic activity comparison after long-term stability. **e** ICP-OES analysis of vanadium in 1.0 M KOH solution during the 110 h HER measurement. **f** EIS spectra of the prepared electrodes. All the electrochemical results were carried out in 1.0 M KOH.

The catalytic performance of Ni(Cu)VOx, Ni(Cu), NiVOx, Ni, and Pt/C (20 wt% Pt on carbon black) for HER was evaluated by using linear sweep voltammetry (LSV) and chronopotentiometry. The electrodeposition bath composition was optimized according to the HER performance (Supplementary Figs. 9 and 10). The best Ni(Cu)VOx HER electrode was prepared by electrodeposition at –2.0 V (vs. SCE) in an electrolyte bath containing 0.5 M NiSO_4_, 12.5 mM CuSO_4_, 7 mM NH_4_VO_3_, and 0.5 M H_3_BO_3_. The doped Cu and V were determined to be Cu/Ni = 0.114 and V/Ni = 0.029 (molar ratio) by inductively coupled plasma optical emission spectroscopy (ICP-OES). In order to confirm that the active materials loaded on FTO and NF are the same, we tested the Ni(Cu)VOx powder scraped from NF by ICP-OES. The doped Cu and V were determined to be Cu/Ni = 0.120 and V/Ni = 0.034 (molar ratio), respectively. This is close to that on FTO. In addition, the molar ratio of Cu/Ni = 0.168 for Ni(Cu) and V/Ni = 0.036 for NiVOx have been determined by ICP-OES. These values are slightly higher than that in Ni(Cu)VOx, suggesting a small change in composition of Ni, Cu and V elements as a result of the electrodeposition process. Shown in Fig. 2a, the Ni(Cu)VOx electrode displays a near-zero onset overpotential for HER and a small overpotential of 21 mV to achieve –10 mA cm^−2^ (without iR correction), which is much smaller than that for Ni(Cu) (49 mV), NiVOx (49 mV), and Ni (87 mV), and comparable to commercial 20% Pt/C catalyst (15 mV). For comparison purpose, the iR-compensated LSV is also displayed (the black dash line in Fig. 2a). An overpotential of merely 10 mV is required to deliver –10 mA cm^−2^, which is the smallest among recently reported non-precious-metal HER catalysts in 1.0 M KOH to our knowledge (Supplementary Table 3). The Tafel slopes obtained for Ni(Cu)VOx, Ni(Cu), NiVOx, Ni, and Pt/C are 28, 72, 45, 74, and 25 mV dec^−1^, respectively (Fig. 2b). The small Tafel slope of 28 mV dec^−1^ for Ni(Cu)VOx suggests the fast kinetics of HER, which follows Volmer-Tafel mechanism with the chemical recombination of adsorbed H species (*H*_ads_) being the rate-determining step. Collectively, these results rank the Ni(Cu)VOx among the most active transition-metal-based electrocatalysts for HER in alkaline medium. Further, to study the interaction effect between Cu and V on HER, the HER performances of Cu and CuVOx were measured under the same conditions and both electrodes display similar HER activity. Of note, the CuVOx electrode shows even decreased activity than the Cu electrode (Supplementary Fig. 11), which demonstrates the interaction of Cu and VOx is not the reason for the improved HER activity.

Electrocatalytic stability is an important criterion for practical applications. As shown in Fig. 2c, with continuous HER at –100 mA cm^−2^, the Ni(Cu)VOx electrode remains relatively stable for more than 100 h after a small increase in overpotential at the start of electrolysis. This behavior is in stark contrast with the poor stability of Ni(Cu) electrode measured at the same condition, where the overpotential increased by more than 230 mV, accounting for 109% overpotential increase (Fig. 2d). The durability of the catalyst is also supported by the post-electrolysis characterizations. The TEM images show Ni(Cu)VOx keeps its structure and uniform distribution of Ni, Cu, V, and O after 100 h water electrolysis (Supplementary Fig. 12). Moreover, the XRD patterns obtained for the Ni(Cu)VOx sample after stability test match with the pattern before the test (Supplementary Fig. 13). The concentrations of Ni and Cu in the electrolyte are 0.08 and 0.04 mg mL^−1^ after stability test, indicating there is no dissolution or peeling off Ni and Cu in the catalyst during the long-term stability test. But, the V2p XPS signals decreased to some extent, which can be attributed to the partial dissolution of the physically absorbed or weakly bonded VOx with Ni in the composite that dissolved in alkaline electrolyte and thus leads to the reduced activity^28,29^ (Supplementary Figs. 14 and 15). However, the concentration of the dissolved VOx in the electrolyte remains constant after 80 h water electrolysis, which is confirmed by the ICP-OES (Fig. 2e). This results in the unchanged overpotential afterwards. The generated H_2_ gas was qualified by gas chromatography (GC) and a Faradaic efficiency of 99.5 ± 1% is obtained (Supplementary Fig. 16 and Supplementary Note 2).

### Electronic and atomic structure analysis

Further mechanistic studies were carried out to reveal the origin of the outstanding HER performance on Ni(Cu)VOx. Firstly, the charge transport properties were analyzed by electrochemical impedance spectroscopy (EIS). Fig. 2f shows Cu doping can accelerate the electron transfer during HER and the charge transfer resistance (*R*_ct_) in Ni(Cu)VOx (*R*_ct_ = 9.4 Ω) is much smaller than that of the control samples, revealing fast charge transfers and minimal parasitic ohmic losses during HER. Secondly, the electrochemical surface area (ECSA) was evaluated using double-layer capacitance (*C*_dl_), determined from CV at different scan rates^30^. The *C*_dl_ value of Ni(Cu)VOx is the highest among the tested samples (Supplementary Figs. 17 and 18 and Supplementary Note 3). After considering the surface area effect by normalizing the current against ECSA, the Ni(Cu)VOx electrode still shows highest HER activity (Supplementary Fig. 19), indicating the intrinsic catalytic activity of each active site is enhanced as a result of VOx modification. Apart from ECSA, we also measured the Brunauer–Emmett–Teller (BET) specific surface area of Ni, Ni(Cu), NiVOx and Ni(Cu)VOx samples with 17.988, 26.614, 18.994, and 34.232 m^2^ g^−1^, respectively (Supplementary Fig. 20 and Supplementary Note 4). As shown, the Ni(Cu)VOx still shows the largest specific surface area.

**Fig. 3:**
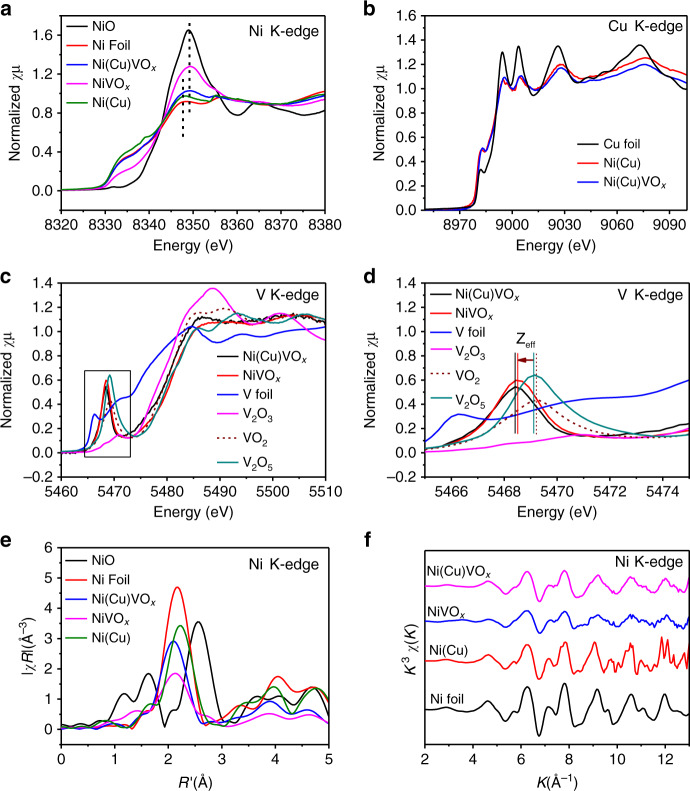
X-ray absorption spectroscopy fine structures. **a** Ni and **b** Cu K-edge XAS. **c**, **d** K-edge XANES on V and the enlarged area marked in **c** *Z*_eff_ = Effective nuclear charge. **e** FT-EXAFS and **f** EXAFS of the samples on Ni K-edge.

In order to determine the elemental oxidation states and the influence of VOx on Cu-doped Ni for HER, Ni, Cu, and V K-edge XAS were carried out and examined at the X-ray absorption near edge structure (XANES) and extended X-ray absorption fine structure (EXAFS) regions. Fig. 3a shows the Ni K-edge spectra of the samples compared to Ni foil and NiO references. Adding VOx to the structure leads to a notable shift of the Ni K-edge positions towards higher energy, indicating that on average the material is more oxidized. These observations suggest that the VOx can partially oxidize the Ni lattice or introducing an oxidized Ni component to the lattice. The XANES spectra of the materials at the Cu K-edge indicate that copper is close to Cu^0^ (Fig. 3b). The materials were also studied at the V K-edge, which is particularly informative in terms of understanding the structure. The V K-edge XANES data of these materials were compared to vanadium oxide reference compounds of different V oxidation states including V_2_O_3_ (3+), VO_2_ (4+), and V_2_O_5_ (5+). As shown in Fig. 3c, d, the intense pre-edge feature and edge position of both Ni(Cu)VOx and NiVOx suggest vanadium has an oxidation state between 4+ and 5+, consistent with the XPS results (Supplementary Fig. 7). It should be noted that it is not accurate to interpret oxidation state from the position of the XANES alone. This is because the likely coordination numbers (4 coordinate vs. 5 coordinate) can also impact the XANES position, as can the nature of the bonding, for example OH^−^ vs. O^2−^. For this reason, in our catalysts, we make a point to not over interpret the data and leave the assignment of the V oxidation state as a range of 4+/5+. Interestingly, the pre-edge position is shifted to lower energy relative to the reference VO_2_ and V_2_O_5_ compounds (Fig. 3d, arrow), indicating a shift in either effective nuclear charge or a change in ligand field^31,32^. As ligand field change usually affects the spectral shape in addition to the energy shift in an open shell system, the most likely reason for the shift is that the metallic Ni environmental influences the effective nuclear charge at the VOx sites^31,32^. Moreover, in Fig. 3d, the position changes are consistent with a change in *Z*_eff_. These are well above experiment resolution and provide unambiguous evidence for the presence of Ni–O–VOx interaction.

The direct Ni–O–VOx interaction was further evidenced by comparing the electrodeposited pure VOx material with Ni(Cu)VOx and NiVOx. As seen from Supplementary Fig. 21a and Supplementary Note 5, the effective nuclear charge of the Ni(Cu)VOx and NiVOx relative to VOx is also observed at lower energy. In addition, we have also collected the XPS data for VOx, Ni(Cu)VOx, and NiVOx. As seen from Supplementary Fig. 21b, compared with Ni(Cu)VOx and NiVOx, the characteristic peak of pure VOx located at a higher binding energy position, which, on the one hand indicates pure VOx has a higher valence state, and on the other hand means there is a direct interaction between Ni and VOx in Ni(Cu)VOx and NiVOx.

To better understand the structural implications of adding VOx to Ni(Cu), EXAFS analysis was performed at the Ni, V, and Cu K-edge. The Fourier transform (FT) and the EXAFS at Ni K-edge data show that the introduction of VOx causes a substantial dampening to the Ni K-edge EXAFS collected on both Ni(Cu)VOx and NiVOx samples (Fig. 3e, f), indicating that the presence of VOx introduces some types of disorder to the metallic Ni lattice. This is in agreement with XRD (see above). But, as seen from XPS spectra (Supplementary Fig. 7), there are abundant Ni^2+^ in Ni(Cu)VOx, while no distinct first-shell of Ni–O is observed in Ni K-edge FT-EXAFS. This is due to that XPS is a surface technique and that XAS in the configuration used in these experiments is a bulk technique. However, as can be seen from Fig. 3a in the Ni K-edge, the white line intensity and its peak position indicate Ni in Ni(Cu)VOx was mildly oxidized (more explanations can be found in Supplementary Note 6).

From simulations of the EXAFS at V K-edge, there were no direct V–Ni/Cu bond in Ni(Cu)VOx, but a well fit with a V–O bond was achieved, which is consistent with the interaction between Ni and V as bridged by Ni–O–VOx (see expanded details of the EXAFS fits in Supplementary Notes 6 and 7, Supplementary Figs. 22–24 and Supplementary Tables 1–2). This result further demonstrates that VOx plays a crucial role on modifying the geometric and electronic structure of the composite that explains the enhanced HER activity. It should be noted that in Supplementary Fig. 25a, there is a clear second shell for Ni(Cu)VOx at V K-edge FT-EXAFS, which may be caused by the surface adsorbed VOx. However, the different position of it in comparison with pure VOx and V_2_O_5_ indicates the Ni–O–V interaction. Moreover, it is clear from Fig. 3b and Supplementary Fig. 25b, by comparing between the XAS data, the similar XAS features of Ni(Cu) and Ni(Cu)VOx at Cu K-edge indicate that Cu substitutes the Ni site in Ni(Cu)VOx.

### Theoretical simulations

With the determined structure of the material in hand, firstly, the structure model was created based on experimental observations (Supplementary Fig. 26). Then, to evaluate the effects of vanadium oxide and Cu on the oxidation degree of Ni(111) catalysts, we calculated the adsorption energies of the O adatom ($E_{{\mathrm{ads}}}^{\mathrm{O}}$) on various Ni(111) surfaces and compared with bare Ni(111) surface (Supplementary Fig. 27). Note that test calculations show that *fcc* site is the most energetically stable site for adsorbed O on Ni(111), thus we only considered *fcc* site as the adsorption site in this work. Supplementary Fig. 27a-c shows the calculated $E_{{\mathrm{ads}}}^{\mathrm{O}}$ on bare and doped Ni(111) with the most energetically stable configurations. The values of $E_{{\mathrm{ads}}}^{\mathrm{O}}$ on bare Ni(111), vanadium oxide doped Ni(111) (V-Ni(111)) and vanadium oxide and Cu co-doped Ni(111) (V/Cu–Ni(111)) are 0.15, –0.61, and –0.54 eV, respectively, which follows the order in the sequence of Ni(111) > V/Cu–Ni(111) > V–Ni(111) (more negative formation energy indicates more energetically stable structure and more easily oxidized). These results are in good agreement with the experimental observations that bare Ni(111) is hardly oxidized, V–Ni(111) is easily oxidized, and V/Cu–Ni(111) is in between the two.

**Fig. 4:**
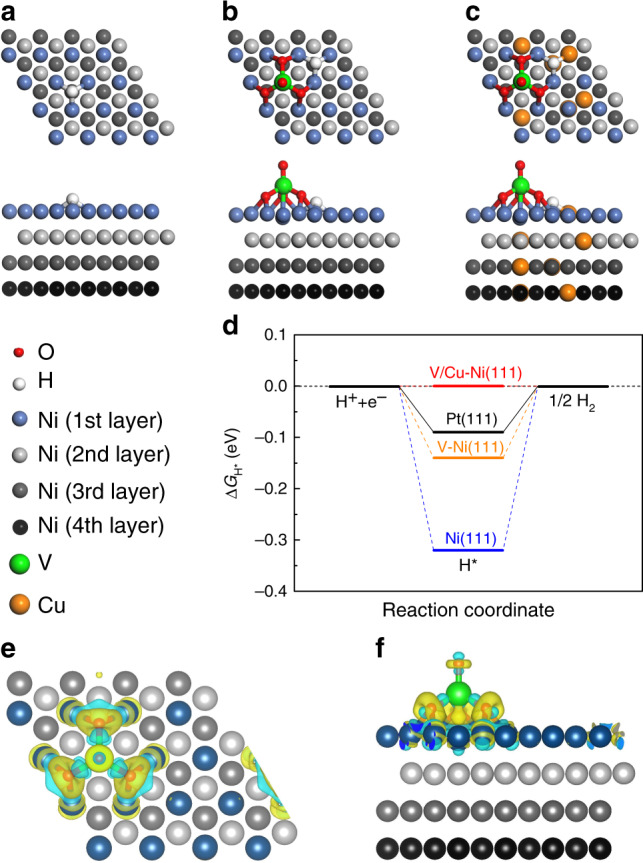
Theoretical calculation of HER activation energy on the prepared catalysts. **a** The optimized structures of H^*^ adsorbed on bare Ni(111), **b** V–Ni(111), and **c** V/Cu–Ni(111). **d** The calculated Δ*G*_H*_ diagram of the HER at the equilibrium potential for various Ni(111) catalysts. The values for Pt(111) is also shown here for comparison. **e**, **f** The deformation electronic density of V–Ni(111). Yellow and cyan refer to electron-rich and electron-deficient area, respectively. The isosurface value is 0.005 e Å^−3^.

Then, the overall HER mechanism was evaluated with a three-state diagram consisting of an initial H^+^ state, an intermediate H^*^ state, and 1/2 H_2_ as the final product. The free energy of H^*^ (${\mathrm{\Delta }}G_{{\mathrm{H}} \ast }$) is proven to be a key descriptor to characterize the HER activity of the electrocatalyst^33^. To understand the vanadium oxide and Cu co-doping effects on HER activity, we performed a series of DFT calculations to investigate the HER activity for bare Ni(111) and various doped Ni(111) catalysts with partially oxidized Ni surfaces. Fig. 4d shows the calculated free energy diagram for HER on bare Ni(111), V–Ni(111), and V/Cu–Ni(111) with the most energetically stable configurations, which are shown in Fig. 4a–c. For the bare Ni(111), the ${\mathrm{\Delta }}G_{{\mathrm{H}} \ast }$ is quite negative (–0.32 eV), which indicate a strong interaction between H^*^ and Ni(111), manifesting poor HER reaction kinetics. Please note that the ${\mathrm{\Delta }}G_{{\mathrm{H}} \ast }$ value on Ni(111) is very close to the ${\mathrm{\Delta }}G_{{\mathrm{H}} \ast }$ = –0.38 eV on NiO/Ni(111) (Supplementary Fig. 28). Introducing oxidized V cluster VO_4_ on Ni(111) significantly increase the value of ${\mathrm{\Delta }}G_{{\mathrm{H}} \ast }$ to –0.14 eV (Supplementary Fig. 29 and Fig. 4d), suggesting an enhanced HER activity compared to bare Ni(111). However, doping NiVOx with Cu leads to the ${\mathrm{\Delta }}G_{{\mathrm{H}} \ast }$ value approaching to 0 eV, compared to the ${\mathrm{\Delta }}G_{{\mathrm{H}} \ast }$ = –0.14 eV of NiVOx. The reason is because on Ni(111) or V-Ni(111) surface the adsorbed H* bounds to three Ni atoms (Fig. 4a, b), while on V/Cu-Ni(111) surface the adsorbed H* bounds to two Ni atoms and one Cu atom (Fig. 4c). As binding energy of H* on Ni is stronger than that on Cu, the substitution of Cu for Ni surface can increase the value of ${\mathrm{\Delta }}G_{{\mathrm{H}} \ast }$ close to 0 eV. We also did DFT calculations of the ${\mathrm{\Delta }}G_{{\mathrm{H}} \ast }$ values for Ni in different positions of the Ni(Cu)VOx model, as can be seen from Supplementary Fig. 29 and Fig. 30. For NiVOx and Ni(Cu)VOx, the active sites are both at site 1, as it has the optimized ${\mathrm{\Delta }}G_{{\mathrm{H}} \ast }$ values. The deformation electronic density calculation (Fig. 4e, f) and Bader analysis show that the VO_4_ cluster has led to an increase in electronic charge density on VO_4_ and a loss of electron charge density on the surrounding three Ni atoms, and there is 0.22 electron transferred from each Ni to VO_4_ based on the Bader analysis. This is consistent with the experimental observations in XAS, where lowered effective nuclear charge is seen at the V K-edge. Less electrons localized on Ni sites closest to VO_4_ results in a closer optimal value of $\left| {{\mathrm{\Delta }}G_{{\mathrm{H}} \ast }} \right|$ on Ni(111), i.e., the enhanced HER activity. In addition, as seen from Fig. 1e–h the TEM EDS mapping and the XPS depth profiling (Supplementary Fig. 31), VOx are distributed uniformly in the composite. Thus, we constructed a DFT model of Ni(111) doped with VO_3_ cluster inside Ni lattice (Supplementary Fig. 32) to simulate the case that VOx located in Ni lattice. We find that the ${\mathrm{\Delta }}G_{{\mathrm{H}} \ast }$ at site 1 is –0.24 eV, which is less negative than that on bare Ni(111) (–0.32 eV). This indicates that VOx located inside Ni lattice can also enhance the HER activity. However, compared to the VOx doped on Ni(111) surface, their contribution to HER enhancement is very small. It is also noted that ${\mathrm{\Delta }}G_{{\mathrm{H}} \ast }$ at other sites for Ni(111) with VO_3_ cluster inside Ni lattice are comparable or more negative (–0.32 ~ –1.16 eV) than that on bare Ni(111) (–0.32 eV). Therefore, VOx is uniformly doped into Ni lattice but the surface VOx contributes mostly to the enhanced HER. Clearly, the V and Cu co-doping generates synergistic effects to improve the electrocatalytic activities towards HER. The HER activity as calculated by DFT follows the order in the sequence of V/Cu–Ni(111) > V–Ni(111) > Ni(111), which are well consistent with experimental results.

## Discussion

To further demonstrate the interaction between Ni and VOx and its effect on HER, we have tried to perform the in situ Raman spectroscopy at an applied negative potential of –1.2 V (vs. Ag) under HER condition by mounting the Ni(Cu)VOx electrode onto a screen-printed electrode. However, the generated hydrogen bubbles have severely affected the laser beam and thus no signals can be collected. Therefore, we applied a semi in situ approach to measure our sample. That is, the Raman spectra were collected immediately after 20 min HER reaction. As seen from Supplementary Fig. 33, compared with the Ni(Cu) electrode, the shift of the Raman spectra for Ni–O bind indicates a direct interaction between VOx and Ni. For the Ni(Cu)VOx Raman spectra collected at open circuit potential and –1.2 V (vs. Ag), there is a clear decrease on peak intensity for V–O at 800–900 cm^−1^, which indicates the dissolution of the physically absorbed and weakly bonded VOx. In addition, there is also a slight peak decrease for Ni–O, which can be attributed to the reduction of Ni–O at negative potential. However, the interactions between VOx and Ni are still existed, which is responsible for the enhanced HER activity, as evidenced from XAS and DFT calculations.

Based on the above results, a reaction mechanism is proposed for HER at the Ni(Cu)VOx catalyst. The modification of Ni(Cu) with VOx has led to the generation of superactive Ni–O–VOx sites with larger electrochemical active surface area, and partial electron transfer from Ni to VOx. This results in the weakened H-adsorption on Cu-doped Ni(111) and thus the enhanced HER activity. Noted that Cu-doped Ni has advantage over Ni, because of the weaker binding of H* on Cu compared to that on Ni^34^. After exposing the Ni(Cu)VOx electrode to KOH electrolyte for HER, the physically absorbed and weakly bonded VOx on the electrode surface is dissolved into the electrolyte (Fig. 2e)^28,29^, causing a slightly decreased HER activity at the beginning of the electrolysis (Fig. 2c). However, the robust stability is observed after the initial activity decrease, because the existed steady Ni–O–VOx sites also favors the H_2_ desorption to refresh the active surface, as revealed by DFT calculation.

In summary, a facile electrochemical deposition approach is developed for preparation of Ni(Cu)VOx electrode with implanted superactive Ni–O–VOx sites for efficient and stable alkaline water splitting. Experimental and theoretical results show that the formed Ni–O–VOx active sites have a profound effect on both tailoring the atomic and electronic structure of the composite and thus resulting in suitable H-adsorption and H_2_ desorption free energy on Ni to achieve enhanced HER activity and stability. It is anticipated that this facile, one-step electrodeposition approach can be easily adapted for large-scale electrode production, and for the wide spread application of the water electrolysis technology for hydrogen production.

## Methods

### Materials

All the chemical reagents and solvents were of analytical grade and were used as received from Sigma-Aldrich without any further purification. FTO-coated glass slides were purchased from Dyesol Ltd. All the solutions were prepared using ultrapure deionized water (18.2 M Ω cm^−1^ at 23 °C (Milli-Q)).

### Preparation of Ni(Cu)VOx electrodes

All electrodeposition were carried out in a standard three-electrode electrochemical cell with NF or FTO as the working electrode, a graphite plate as the auxiliary electrode, and a double junction SCE as the reference electrode. Prior to deposition, the NF was firstly ultrasonicated in 5 M HCl solution for 10 min to remove the NiO layer, rinsed subsequently with water and ethanol, and then dried in air. The electrodeposition was conducted in an electrolyte containing 0.5 M NiSO_4_, 12.5 mM CuSO_4_, 7 mM NH_4_VO_3_, and 0.5 M H_3_BO_3_, by dissolving each chemicals in 50 mL Milli-Q water under sonication and the electrodeposition then was carried out with a CHI 760D electrochemical workstation at –2.0 V (vs. SCE) for 600 s at room temperature. Catalyst mass loading on NF is: 3.0 ± 0.2 mg cm^−2^, and the absolute amount of each metal is calculated to be ~ 2.6 mg for Ni, ~0.3 mg for Cu and ~0.1 mg for V in the composite based on the molar ratio determined from ICP-OES.

### Electrochemical measurement

All electrochemical measurements were carried out with a CHI 760D electrochemical workstation. The as-prepared electrode on NF was directly used as the working electrode without further treatments. The exposed surface area of the NF electrode is 0.5 × 0.5 = 0.25 cm^2^. A graphite plate and double junction SCE were used as the counter electrode and reference electrode, respectively. All potentials measured were calibrated to the reversible hydrogen electrode using the following equation:

1 $$E_{{\mathrm{RHE}}} = E_{{\mathrm{SCE}}} + 0.241\,{\mathrm{V}} + 0.059\,{\mathrm{pH}}$$

HER linear sweep voltammetry (LSV) polarization curves were recorded at a scan rate of 5 mV s^−1^. All the HER polarization curves were measured in 1 M KOH without iR compensation unless otherwise stated. For iR compensation, an iR compensation level of 95% was applied by using the automated iR-correction function of the potentiostat^35^. Chronopotentiometric measurement was obtained under the same experimental setup. EIS of samples were measured at 200 mV overpotential in the frequency range of 0.1–100,000 Hz with an amplitude of 10 mV in 1 M KOH electrolyte by using Metrohm Autolab instrument.

### X-ray absorption spectroscopy

V, Ni, and Cu K-edge XAS spectra were recorded on the multiple wiggler XAS beamline 12 ID at the Australian Synchrotron, in operational mode 1 using a Si(111) monochromator with focusing optics. The beam energy was 3.0 GeV and the maximum beam current was 200 mA. Vanadium spectra were calibrated against the first inflection point of V foil 5,465 eV; Ni to the first inflection point of Ni foil 8,333 eV and Cu to the first inflection point of the Cu foil 8,979 eV. Electrodes were analyzed ex situ at room temperature in He filled chamber. In each case the thin electrode films were positioned at 45 degrees to the incoming beam to maximize fluorescence signal. Data on the oxide powder for references were collected in transmission mode using ionization chambers, He-filled for V, and N_2_ filled for Ni and Cu. Metal oxide samples for transmission X-ray experiments were prepared by grinding the sample together with boron nitride and loading into a 1 mm thin Al spacer and sealed with Kapton tape. XAS data was analyzed using a combination of PySpline and Microsoft Excel for background subtractions.

### DFT calculations

All of the spin-polarized DFT calculations were performed using the VASP program^36–38^, which uses a plane-wave basis set and a projector augmented wave method for the treatment of core electrons^37^. The Perdew–Burke–Ernzerhof exchange-correlation functional within a generalized gradient approximation (GGA-PBE) was used in our calculations^39^. For the expansion of wave functions over the plane-wave basis set, a converged cutoff was set to 450 eV. Spin-polarization effect and dipole correction were considered in all cases.

The overall HER mechanism is evaluated with a three-state diagram consisting of an initial H^+^ state, an intermediate H^*^ state, and 1/2 H_2_ as the final product. The free energy of H^*^ (${\mathrm{\Delta }}G_{{\mathrm{H}} \ast }$) is proven to be a key descriptor to characterize the HER activity of the electrocatalyst. An electrocatalyst with a positive value leads to low kinetics of adsorption of hydrogen, while a catalyst with a negative value leads to low kinetics of release of hydrogen molecule. The optimum value of $\left| {{\mathrm{\Delta }}G_{{\mathrm{H}} \ast }} \right|$ should be zero; for instance, this value for the well-known highly efficient Pt catalyst is near-zero as $\left| {{\mathrm{\Delta }}G_{{\mathrm{H}} \ast }} \right|\,\approx\,\sim 0.09\,\,{\mathrm{eV}}$. The ${\mathrm{\Delta }}G_{{\mathrm{H}} \ast }$ is calculated as^33^:

2 $${\mathrm{\Delta }}G_{{\mathrm{H}} \ast } = {\mathrm{\Delta }}E_{{\mathrm{H}} \ast } + {\mathrm{\Delta }}E_{{\mathrm{ZPE}}} - T{\mathrm{\Delta }}S_{\mathrm{H}},$$

where ${\mathrm{\Delta }}E_{{\mathrm{H}} \ast }$ is the binding energy of adsorbed hydrogen, and Δ*E*_ZPE_ and Δ*S*_H_ are the differences in ZPE and entropy between the adsorbed hydrogen and hydrogen in the gas phase, respectively. As the contribution from the vibration entropy of hydrogen in the adsorbed state is negligibly small, the entropy of hydrogen adsorption is ${\mathrm{\Delta }}S_{\mathrm{H}} \approx - \frac{1}{2}S_{{\mathrm{H2}}}$, where *S*_H2_ is the entropy of H_2_ in the gas phase at the standard conditions. Therefore, the ${\mathrm{\Delta }}G_{{\mathrm{H}} \ast }$ value for Ni(111) surface should be Δ*E*_H_ + 0.24 eV.

The structural model of bare Ni(111) was constructed as 4 × 4 periodic supercell (Supplementary Fig. 26a), which contains four atomic layers with the bottom two layers fixed in their respective bulk positions and all the other atoms fully relaxed. Experimental observations of V and Cu co-doped Ni(111) structure indicate that doped V atoms are oxidized and adsorb on Ni(111) surface (V/Ni = 0.029), while Ni atoms are substituted partially by Cu atoms (Cu/Ni = 0.114). In order to simulate the doped Ni(111) catalysts, one VO_4_ cluster is added on Ni(111) surface (V-Ni(111), Supplementary Fig. 26b), and seven Ni atoms are replaced by the doped Cu atoms (V/Cu–Ni(111), Supplementary Fig. 26c). The vacuum space was set to larger than 18 Å in the *z* direction to avoid interactions between periodic images. In geometry optimizations, all the structures were relaxed up to the residual atomic forces smaller than 0.005 eV/Å, and the total energy was converged to 10^−4^ eV. The Brillouin zone was sampled using 3 × 3 × 1 Γ-centered mesh. The deformation electronic density of the V–Ni(111) is defined as

3 $$\Delta \rho \left( {\mathrm{r}} \right) = \rho \left( {\mathrm{r}} \right)_{{\mathrm{V}} - {\mathrm{Ni}}\left( {111} \right)} - \rho \left( {\mathrm{r}} \right)_{{\mathrm{Ni}}\left( {111} \right)} - \rho \left( {\mathrm{r}} \right)_{{\mathrm{VO4}}},$$

where *ρ*(r)_V−Ni(111)_ represent the charge density of the V–Ni(111) system, and *ρ*(r)_Ni(111)_ and *ρ*(r)_VO4_ represent the charge density of the bare Ni(111) and the VO_4_ cluster at the same coordinates as those in the V–Ni(111) system, respectively. Moreover, although our calculations are done in the gas phase, similar to Nørskov’s approach^33^, we believe our calculation results are accurate in describing realistic operating conditions. This is because the solvation effect is very small for HER and can be safely ignored, as well explained and confirmed in previous studies^33,40,41^. Therefore, by including solvent effects in the different systems reported here, we can believe that no appreciable change in activities, compared to those in vacuum.

## Supplementary information

Supplementary Information

Peer Review File

## Data Availability

The data that support the plots within this paper and other findings of this study are available from the corresponding author on request.
