# Supplementary material for: Implanting Ni-O-VOx sites into Cu-doped Ni for low-overpotential alkaline hydrogen evolution
Source: Nat Commun. 2020 Jun 1;11:2720. doi: 10.1038/s41467-020-16554-5 (PMC7264301; doi:10.1038/s41467-020-16554-5)
Supplement: Supplementary file 1 — Supplementary Information [file 41467_2020_16554_MOESM1_ESM.pdf]

Supplementary Information

Implanting Ni-O-VOx sites into Cu-doped Ni for low-overpotential alkaline hydrogen evolution

Li, *et al.*

Supplementary Figures

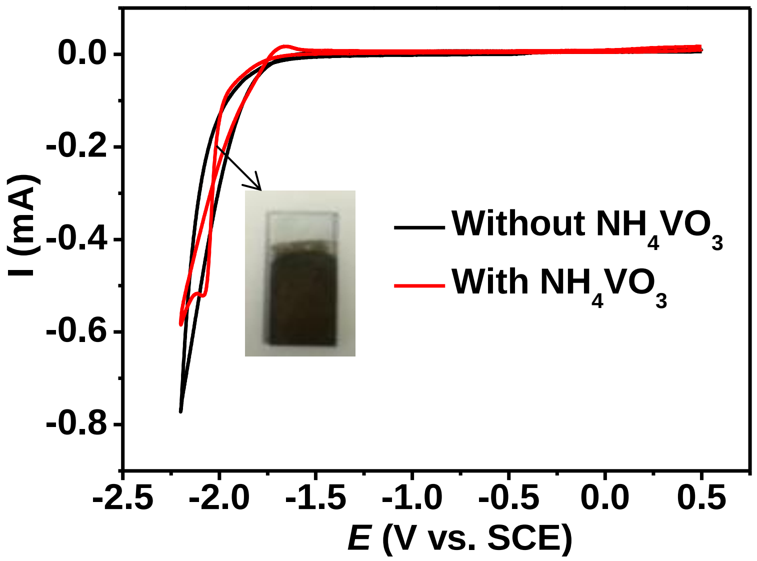

Supplementary Fig. 1 Cyclic voltammetry curves of the VOx sample on FTO. Cyclic voltammetry curves on FTO substrate with present and absent of ammonium metavanadate. Insert: the color of the prolonged electrodeposited vanadium oxide cluster on FTO is black.

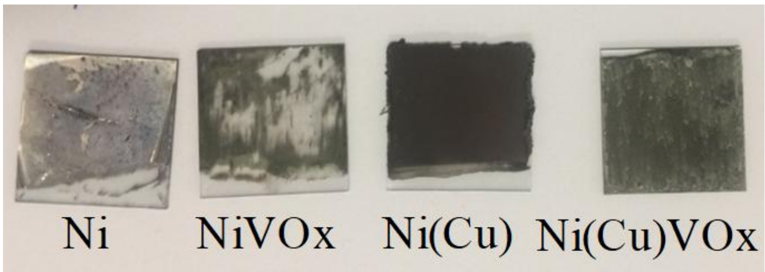

Supplementary Fig. 2 Optical images of the prepared samples. Optical images of the samples electrodeposited on FTO substrate.

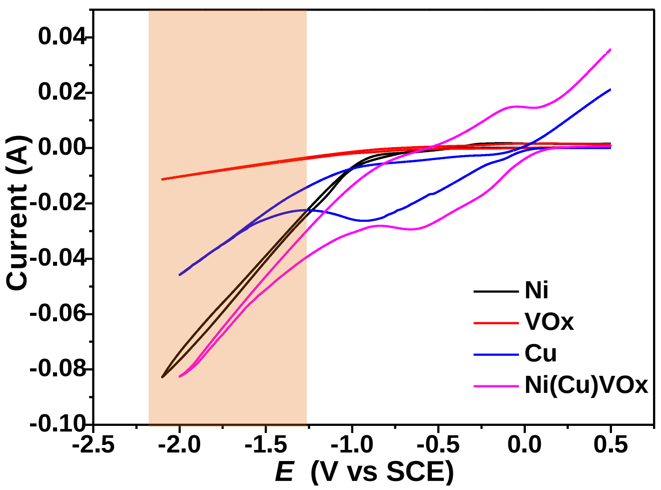

Supplementary Fig. 3 Electrodeposition cyclic voltammetry curves of the prepared catalysts on NF. The cyclic voltammetry curves of electrochemical reduction behaviors of Ni^{2+}, Cu^{2+} and VO^{3-}.

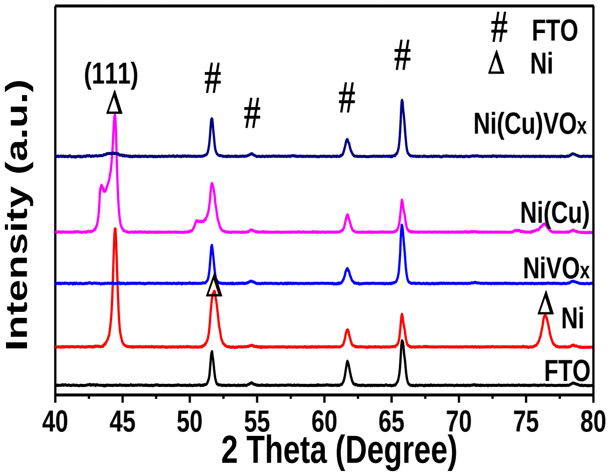

Supplementary Fig. 4 XRD patterns. XRD patterns of the electrodeposited samples on FTO.

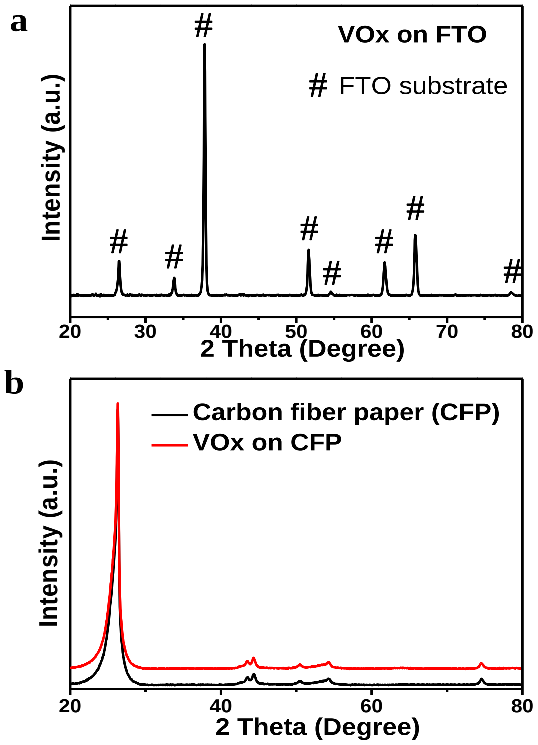

Supplementary Fig. 5 XRD patterns. XRD patterns of the electrodeposited VOx on **a** FTO and **b** CFP substrate.

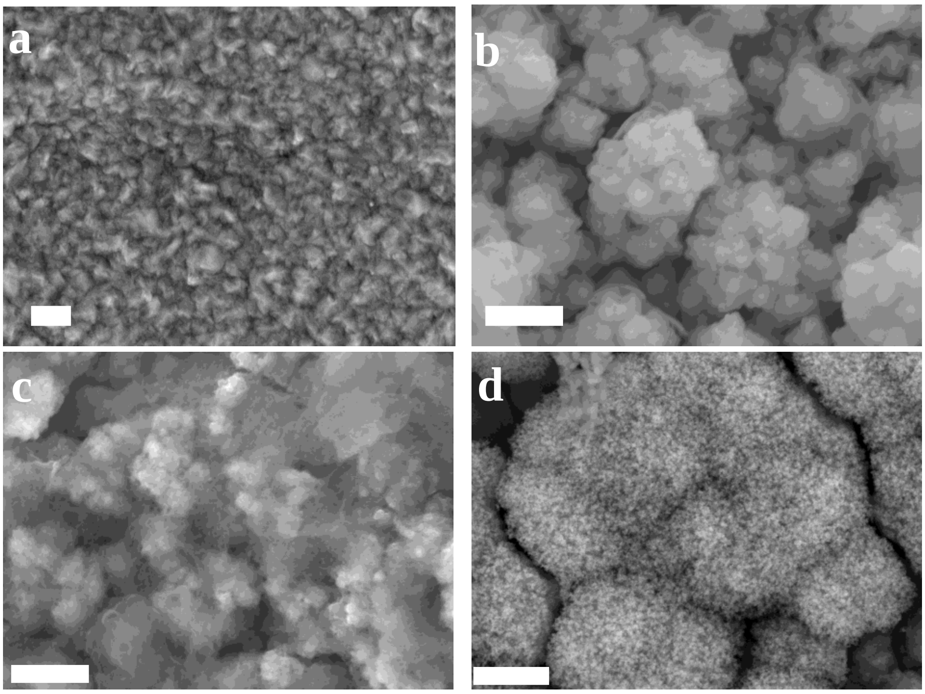

Supplementary Fig. 6 SEM morphology. SEM images for **a** Ni, **b** Ni(Cu), **c** NiVOx, and **d** Ni(Cu)VOx. (scale bar: 1 μm).

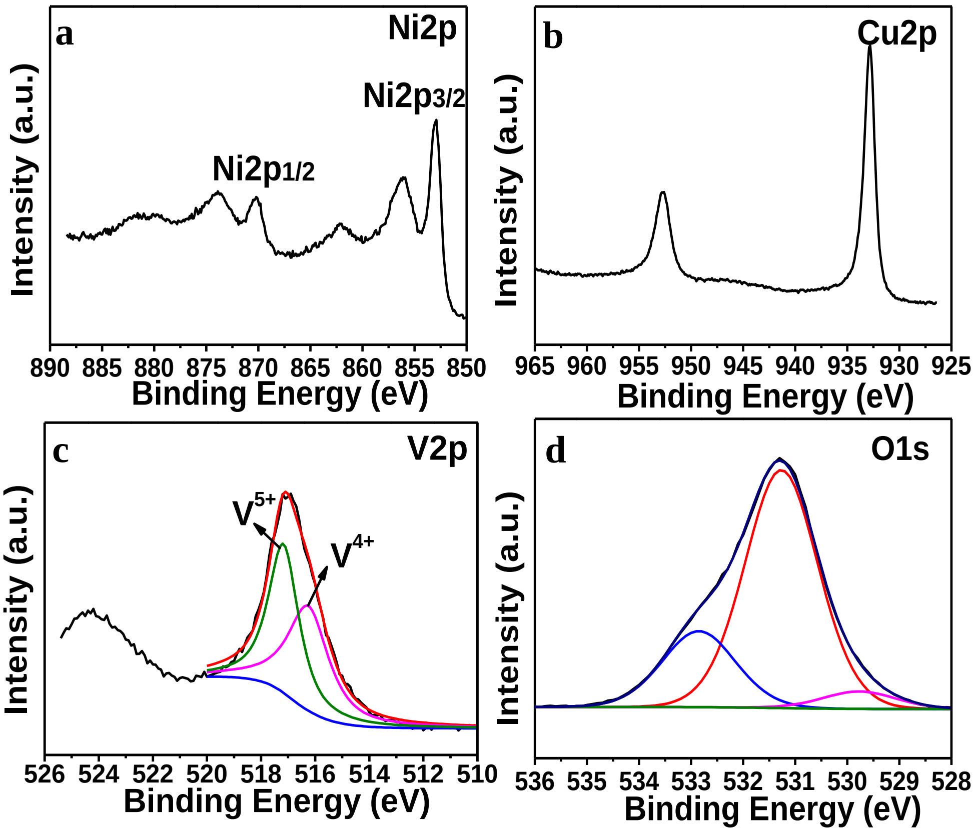

Supplementary Fig. 7 XPS spectra of Ni(Cu)VOx. XPS spectra of Ni(Cu)VOx for **a** Ni2p, **b** Cu2p, **c** V2p and **d** O1s.

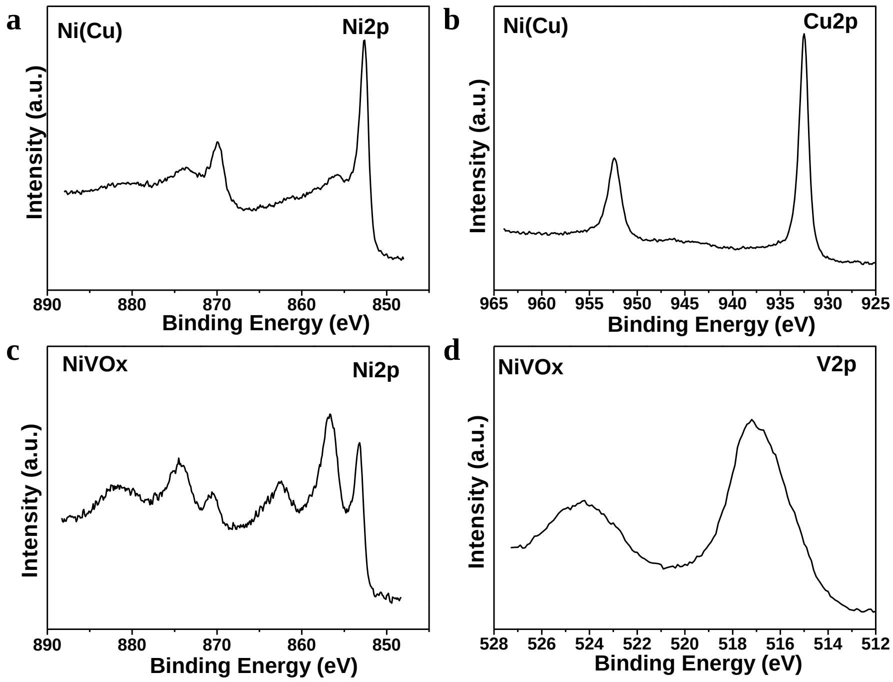

Supplementary Fig. 8 XPS spectra of Ni(Cu) and NiVOx. a Ni2p and **b** Cu2p XPS spectra of Ni(Cu), **c** Ni2p and **d** V2p for NiVOx.

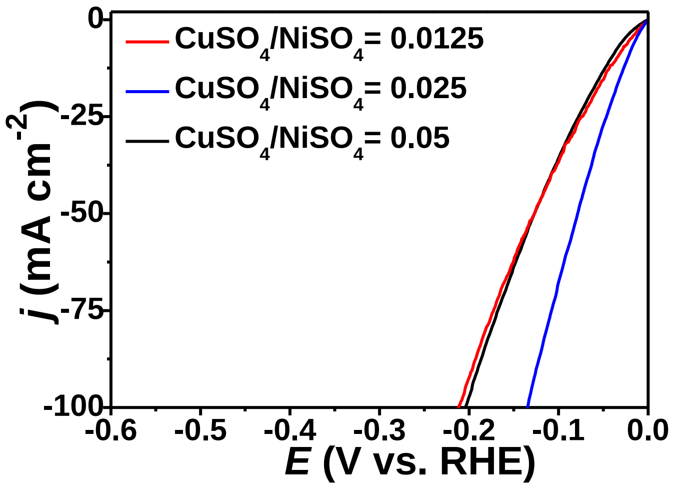

Supplementary Fig. 9 LSV curves for Ni(Cu)VOx. LSV curves in 1 M KOH for Ni(Cu)VOx obtained at different amount of $CuSO_4$ precursor (molar ratio) during electrodeposition.

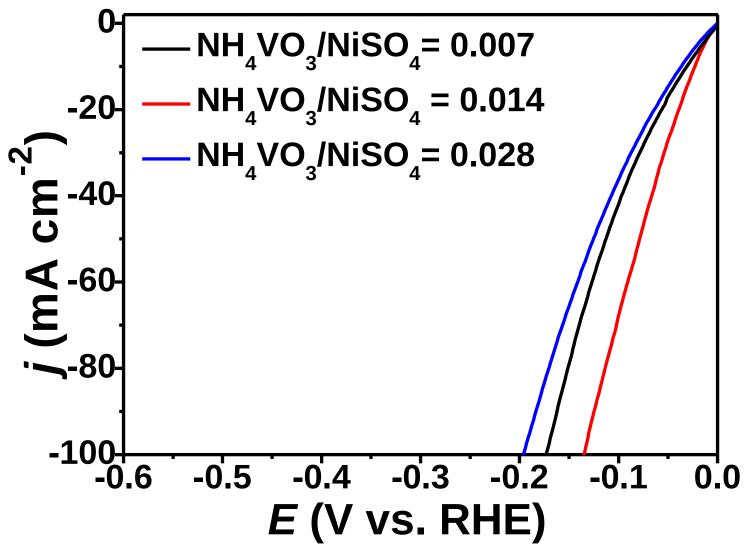

Supplementary Fig. 10 LSV curves for Ni(Cu)VOx. LSV curves in 1 M KOH for Ni(Cu)VOx prepared by using different amount of NH_4VO_3 precursor (molar ratio) during electrodeposition.

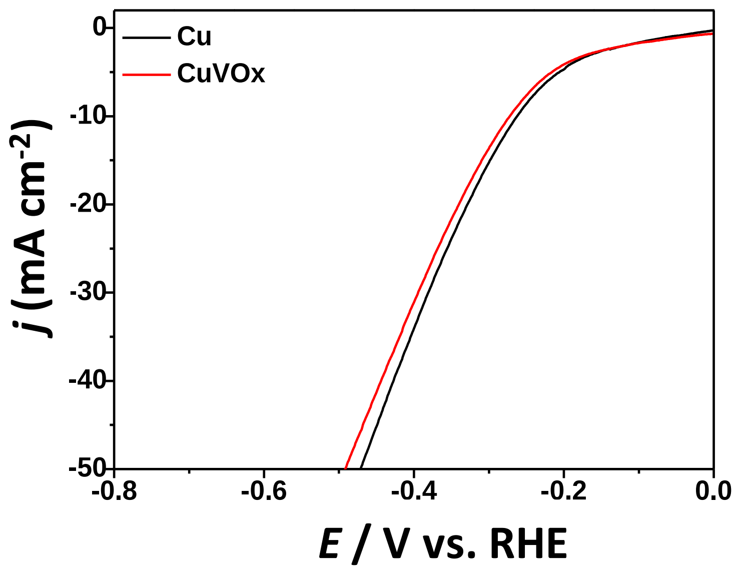

Supplementary Fig. 11 LSV curves for Cu and CuVOx. LSV polarization curves of Cu and CuVOx in 1 M KOH.

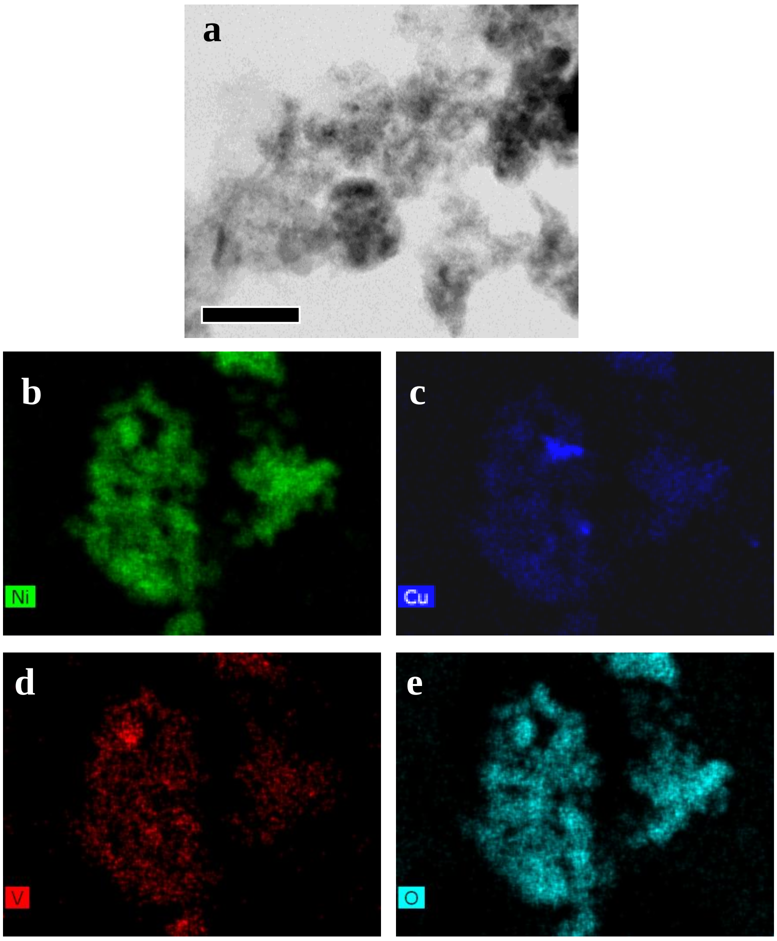

Supplementary Fig. 12 TEM of Ni(Cu)VOx after HER. a TEM and **b-e** TEM-EDS mapping images of Ni(Cu)VOx after HER long-term stability. (scale bar: 50 nm).

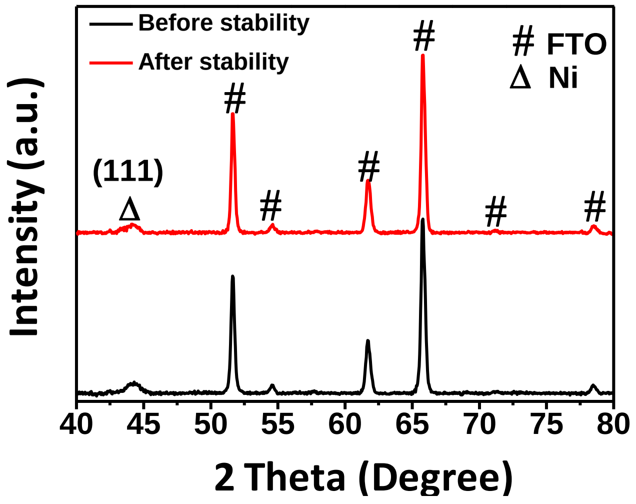

Supplementary Fig. 13 XRD spectra of Ni(Cu)VOx. XRD spectra of Ni(Cu)VOx before and after long-term stability measurement.

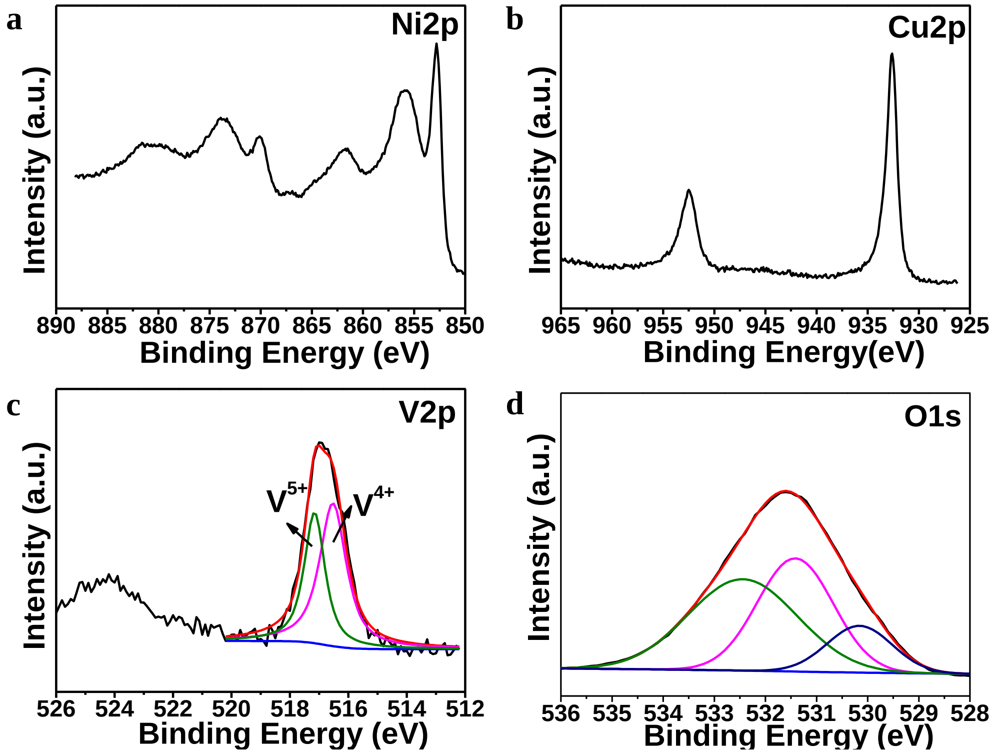

Supplementary Fig. 14 XPS spectra of Ni(Cu)VOx after HER. XPS spectra of Ni(Cu)VOx for **a** Ni2p, **b** Cu2p, **c** V2p and **d** O1s after HER stability.

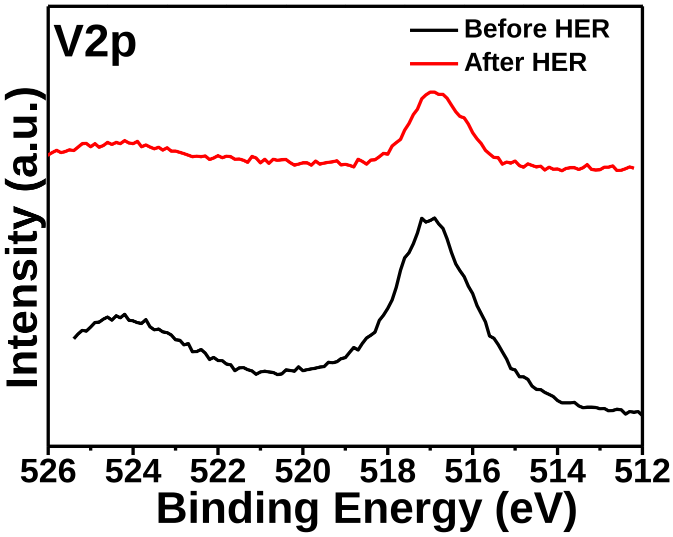

Supplementary Fig. 15 Core-level XPS spectra of V2p. Core-level XPS spectra of V2p for Ni(Cu)VOx, before and after HER stability.

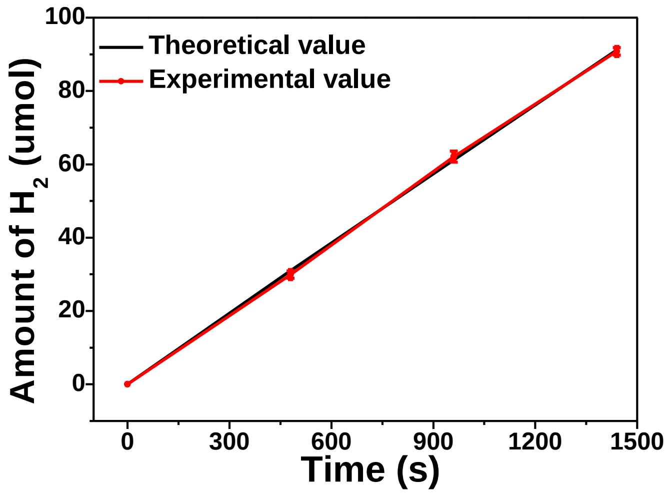

Supplementary Fig. 16 Theoretical *vs*. experimental amounts of H_2 during bulk water electrolysis. The electrolysis was carried out at a typical potential of – 1.2 V (*vs*. SCE) and the generated H_2 is detected online by a gas chromatograph at the electrolysis time of 8 min, 16 min, and 24 min.

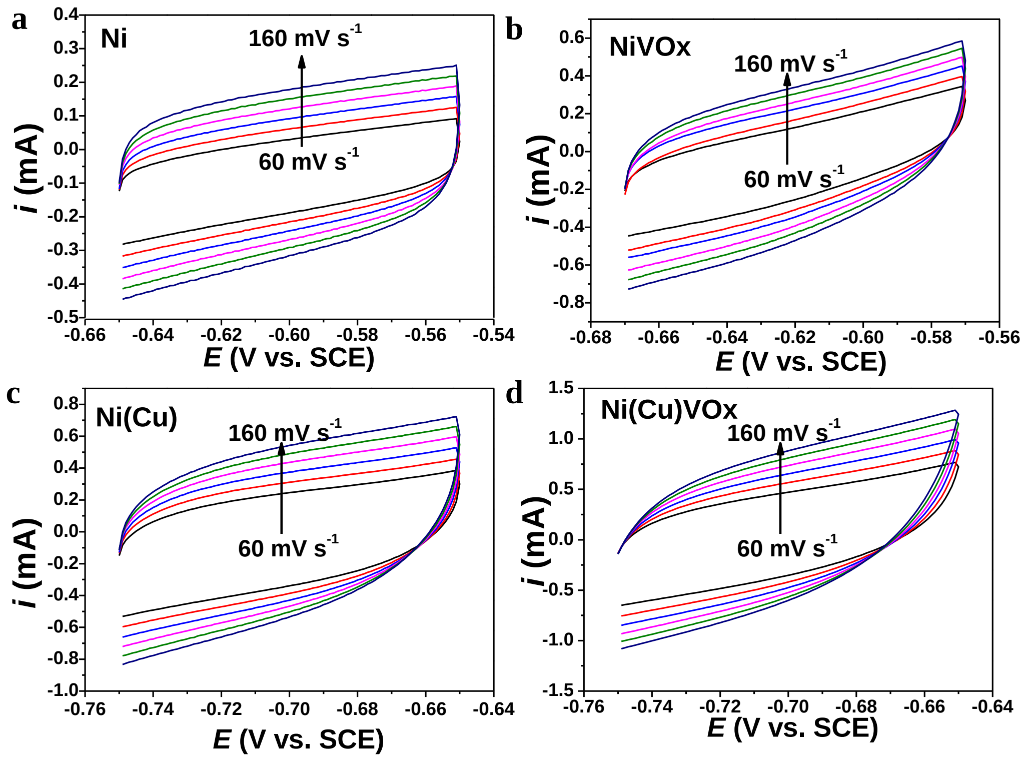

Supplementary Fig. 17 Cyclic voltammograms (CVs) at different scan rates of the prepared samples. a Ni, **b** NiVOx, **c** Ni(Cu) and **d** Ni(Cu)VOx.

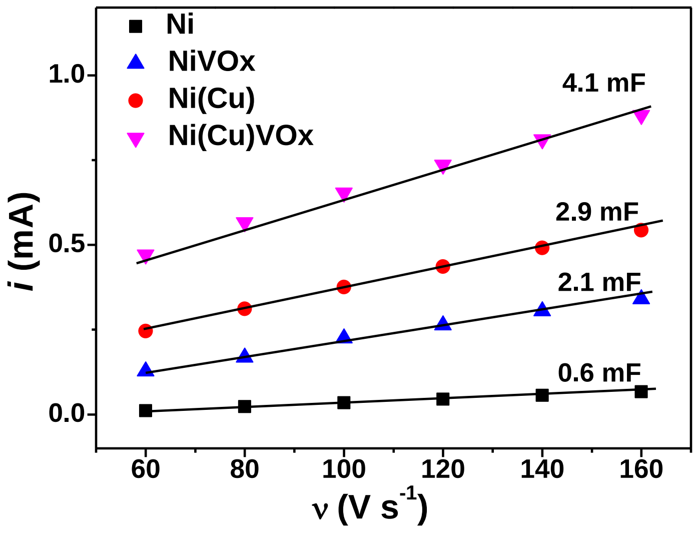

Supplementary Fig. 18 Double-layer capacitance (*C_{dl}*). Double-layer capacitance (*C_{dl}*) of the samples calculated from CVs at different scan rates.

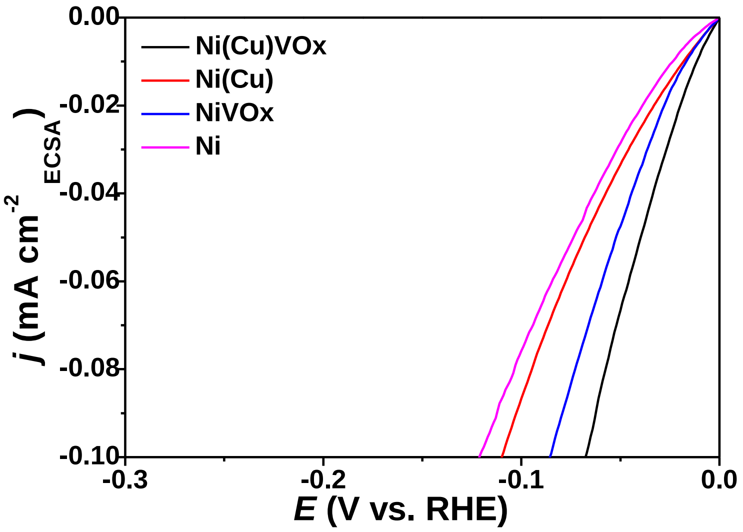

Supplementary Fig. 19 ECSA-normalized HER polarization. ECSA-normalized HER polarization curves of the fabricated electrodes.

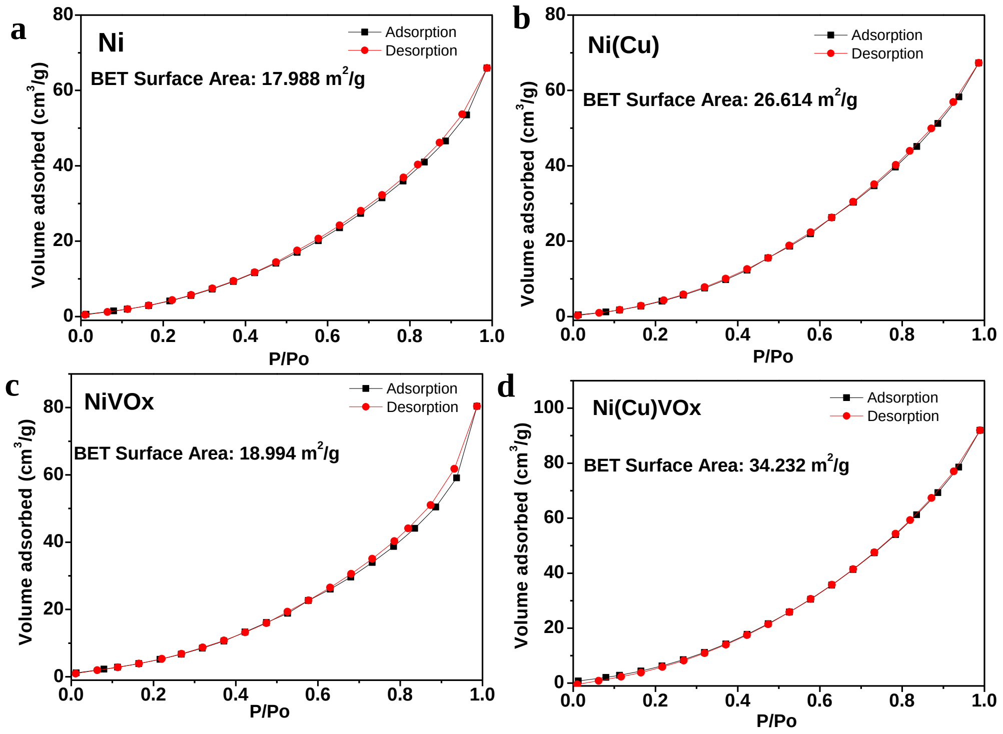

Supplementary Fig. 20 N_2 adsorption/desorption isotherms. N_2 adsorption/desorption isotherms of the prepared catalysts for **a** Ni, **b** Ni(Cu), **c** NiVOx and **d** Ni(Cu)VOx.

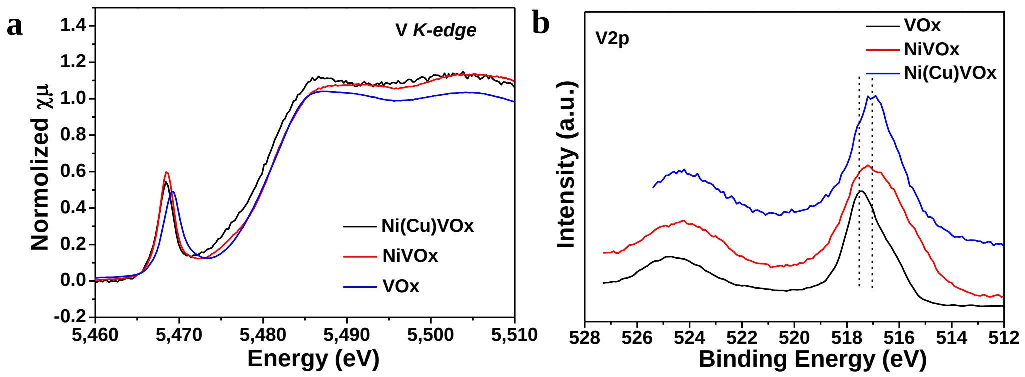

Supplementary Fig. 21 V K-edge XANES and XPS. a V K-edge XANES and **b** V2p XPS spectra of VOx, NiVOx and Ni(Cu)VOx.

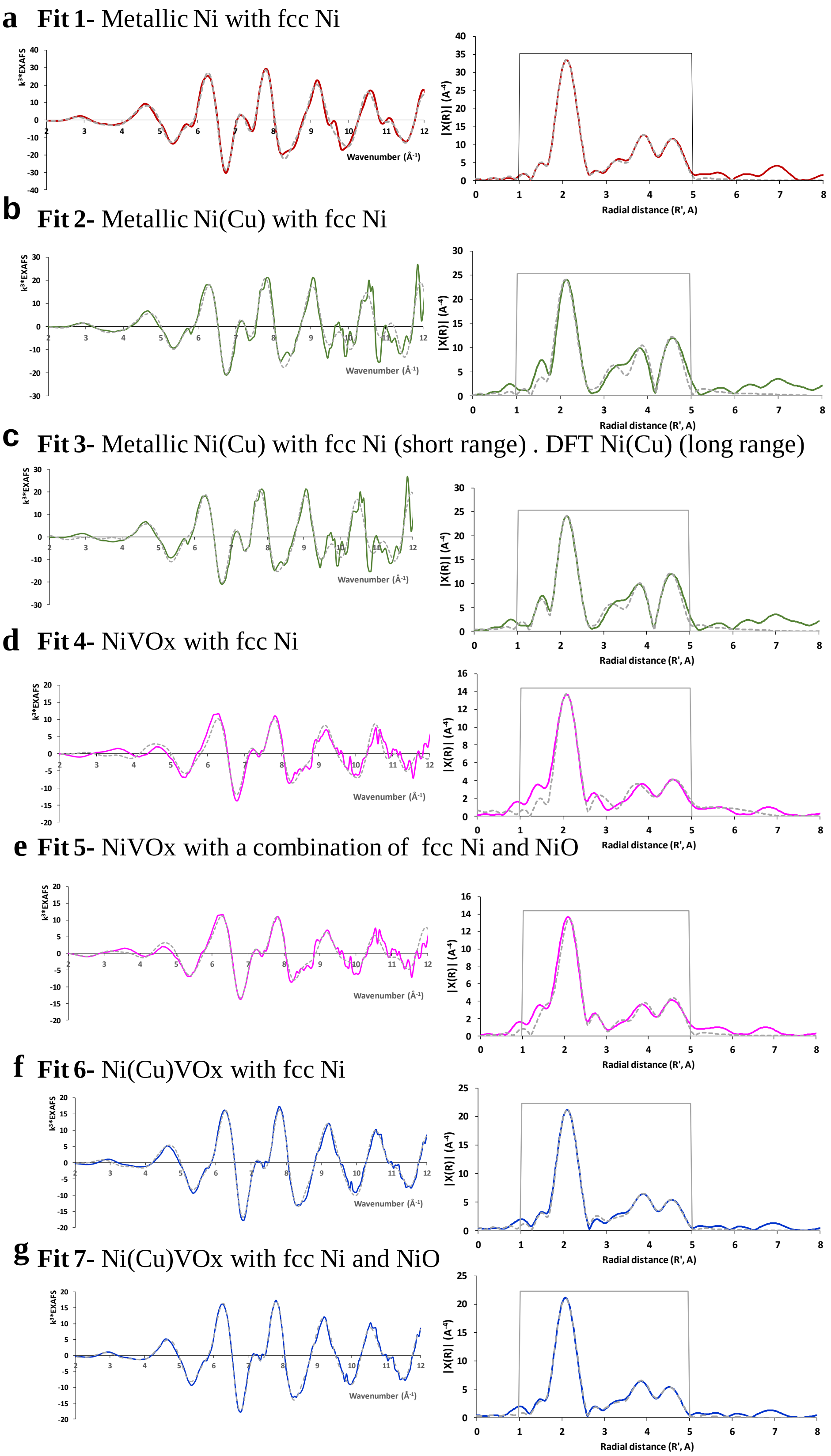

Supplementary Fig. 22 Summary of the EXAFS fits at the Ni K-edge. **a** Ni, **b** Ni(Cu), **c** Ni(Cu), **d** NiVOx, **e** NiVOx, **f** Ni(Cu)VOx and **g** Ni(Cu)VOx.

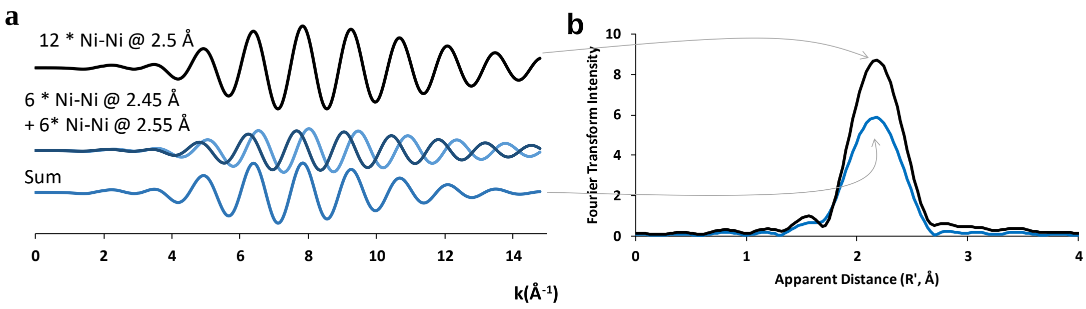

Supplementary Fig. 23 Explanation of disorder disrupts lattice structures. a A small bond length change causes interference to EXAFS paths, which has the effect of dampening the **b** Fourier Transform of the EXAFS. The effect increases with distance and the frequency of the EXAFS contribution.

a. NiVOx

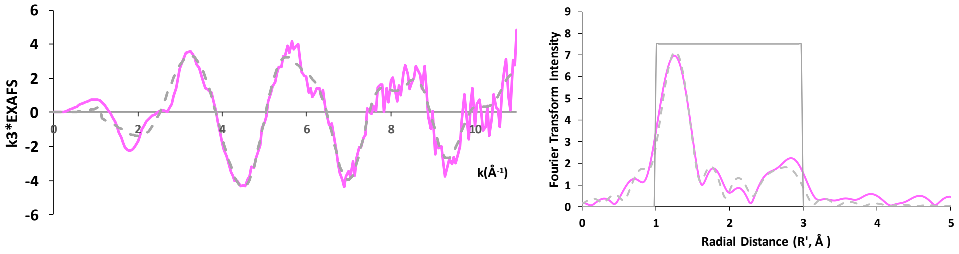

b. Ni(Cu)VOx

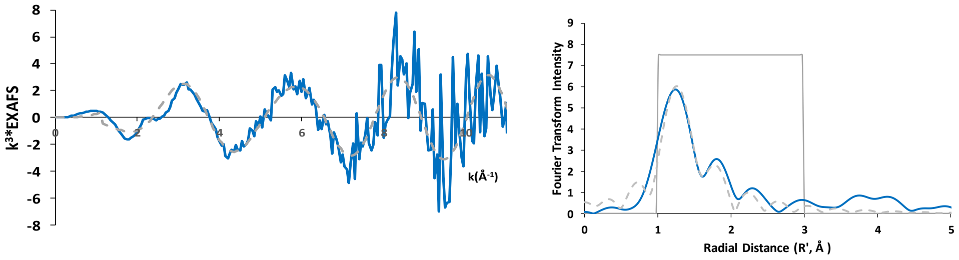

Supplementary Fig. 24 Fit to the V K-edge XAS data. a NiVOx and **b** Ni(Cu)VOx. In each case the experimental data is given in the colored trace and the fit that trace given in the grey trace.

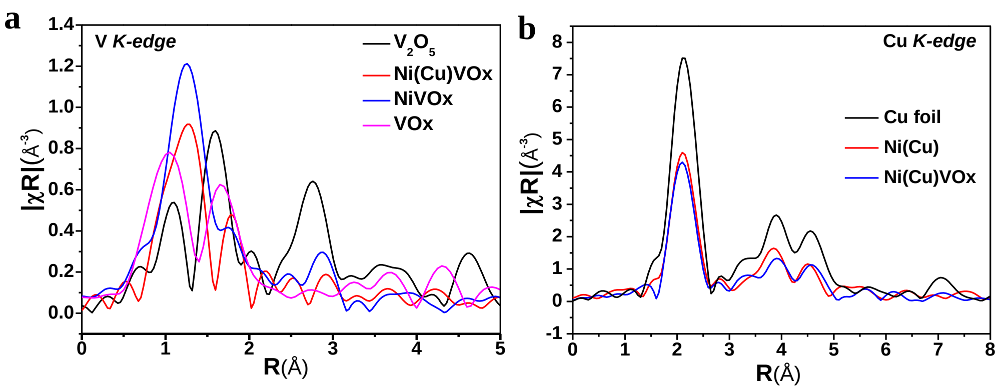

Supplementary Fig. 25 FT-EXAFS. a V K-edge and **b** Cu K-edge FT-EXAFS.

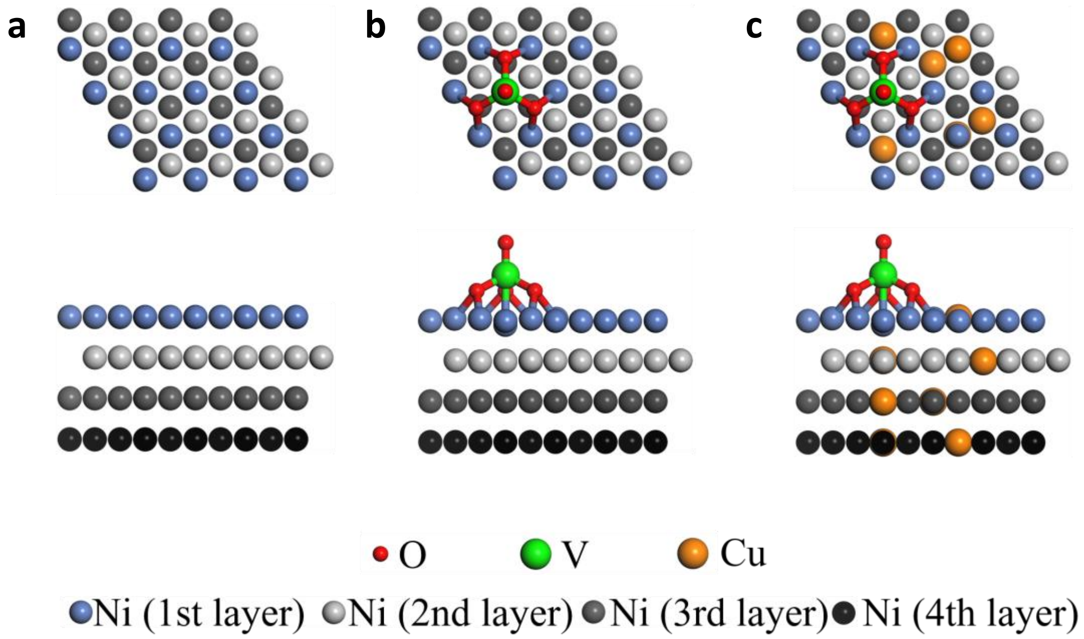

Supplementary Fig. 26 DFT calculations of optimized structures. Top (upper) and side (lower) views of the optimized structures of **a** bare Ni(111), **b** V-Ni(111), and **c** V/Cu-Ni(111).

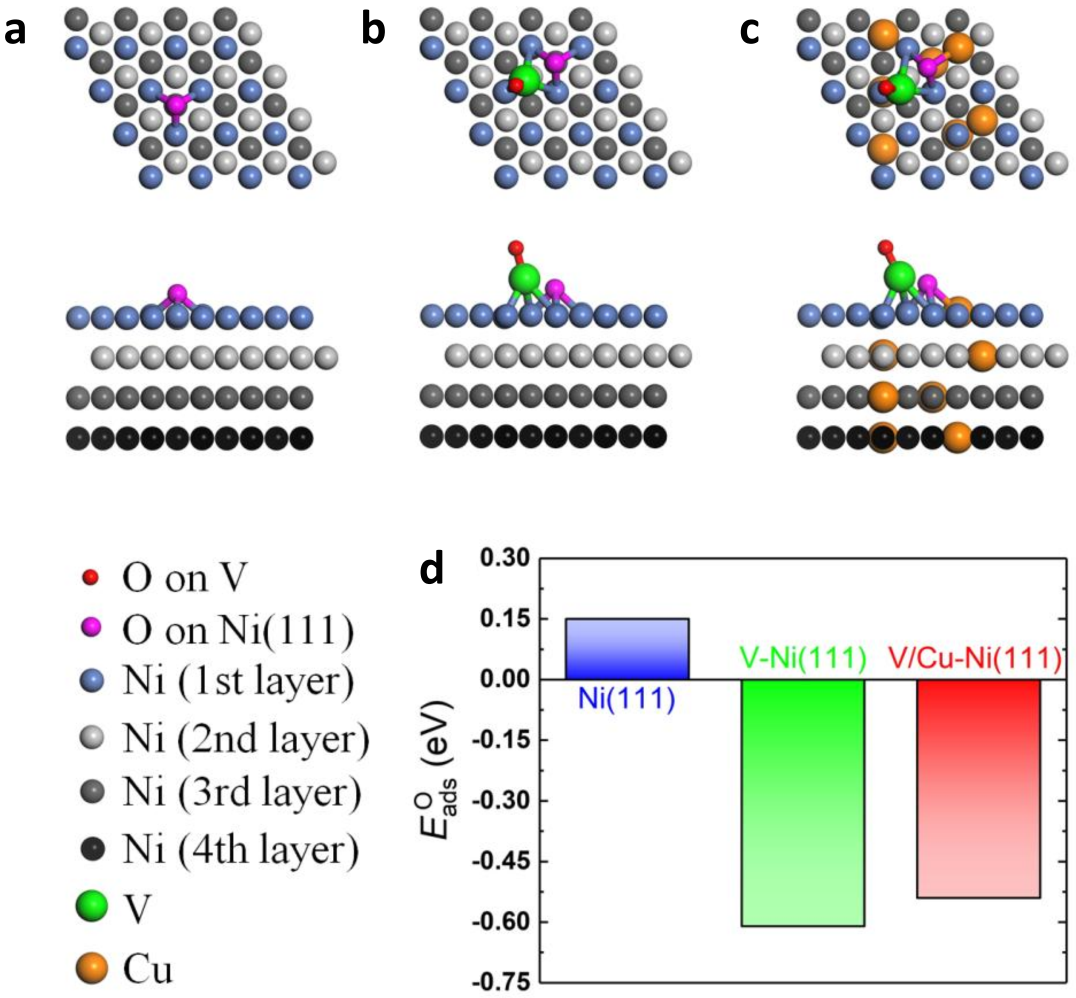

Supplementary Fig. 27 DFT calculations of the optimized structures of the adsorbed O^*. The optimized structures of O^* adsorbed on **a** bare Ni(111), **b** V-Ni(111), **c** V/Cu-Ni(111). **d** The calculated $E_{\text{ads}}^{\text{O}}$ for various Ni(111) catalysts.

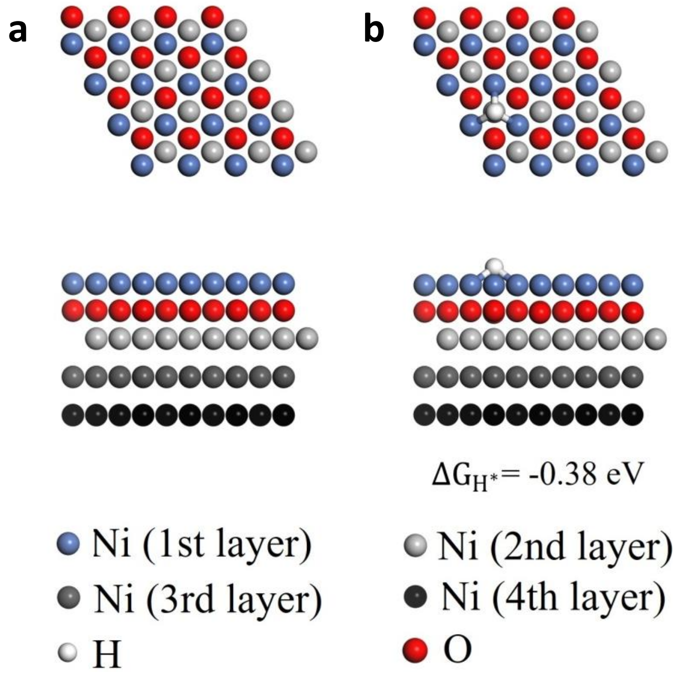

Supplementary Fig. 28 DFT calculations of the optimized structures of NiO/Ni(111). Top (upper) and side (lower) views of the optimized structures of **a** bare NiO/Ni(111) and **b** H^* adsorbed on NiO/Ni(111). The calculated ΔG_{H^*} of H^* adsorbed on NiO/Ni(111) are listed in **b**.

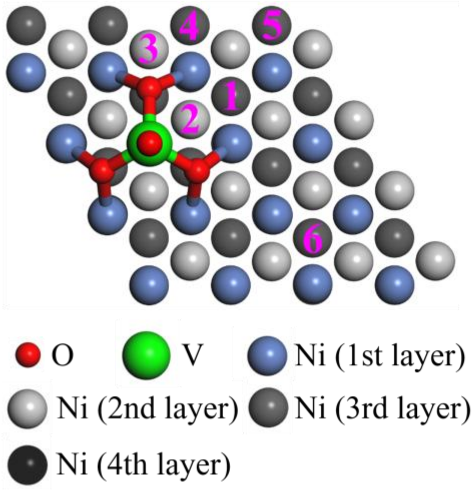

H* Adsorption Site	ΔG_{H^*} (eV)
1	-0.14
2	0.94
3	0.54
4	-0.16
5	-0.29
6	-0.30

Supplementary Fig. 29 Structure of the V-Ni(111) used in our calculations. The magenta numbers denote the possible adsorption sites for H* closed to the oxidized V cluster VO_4, and the corresponding ΔG_{H^*} are listed in the table on the right.

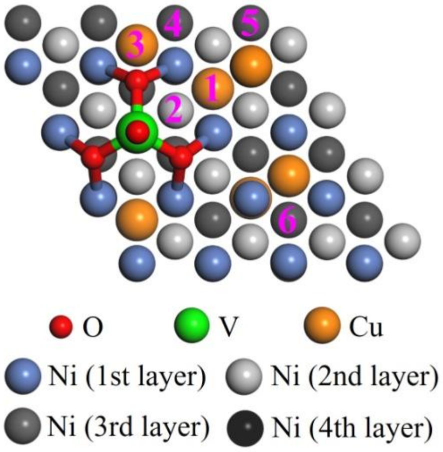

H* Adsorption Site	ΔG_{H^*} (eV)
1	0.00
2	0.89
3	0.26
4	-0.23
5	-0.22
6	-0.31

Supplementary Fig. 30 Structure of the V/Cu-Ni(111) used in our calculations. The magenta numbers denote the possible adsorption sites for H* closed to the oxidized V cluster VO_4, and the corresponding ΔG_{H^*} are listed in the table on the right.

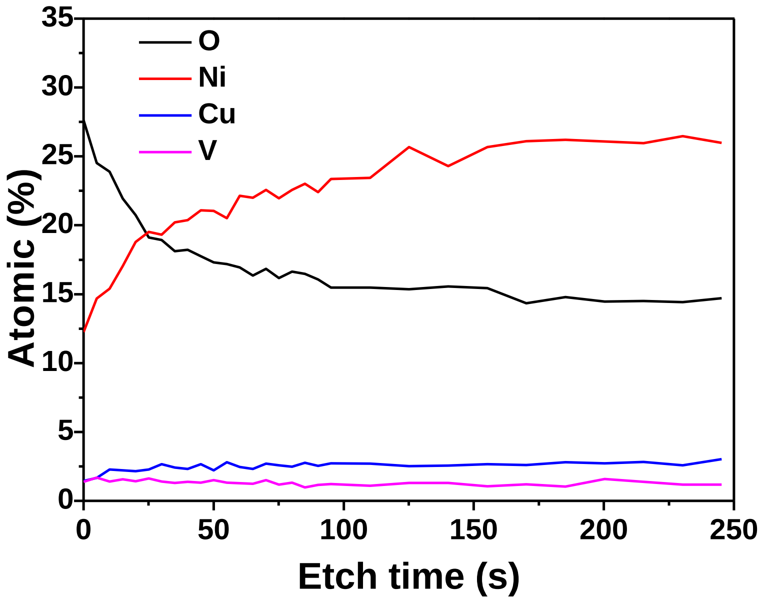

Supplementary Fig. 31 XPS depth profile. XPS depth profile of Ni(Cu)VOx.

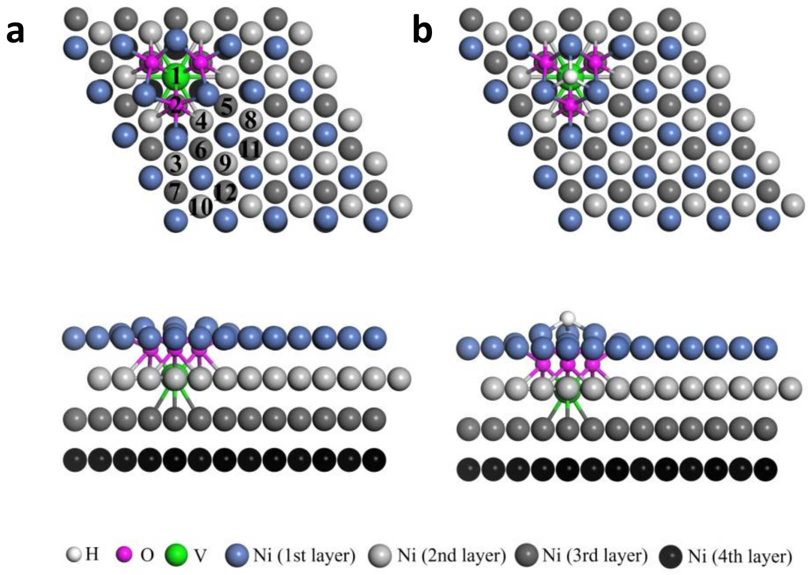

H* Adsorption Site	1	2	3	4	5	6	7	8	9	10	11	12
ΔG_{H^*} (eV)	-0.24	-1.16	-0.55	-0.45	-0.54	-0.39	-0.33	-0.32	-0.32	-0.34	-0.32	-0.35

Supplementary Fig. 32 DFT optimized structure of VO_3 cluster in Ni lattice. a The optimized structure of VO_3 cluster in Ni lattice. **b** The optimized structure of H* adsorbed at site 1 on Ni(111) surface with VO_3 cluster in Ni lattice. The black numbers in **a** denote the possible adsorption sites for H* closed to the oxidized V cluster VO_3, and the corresponding ΔG_{H^*} are listed in the table on the bottom.

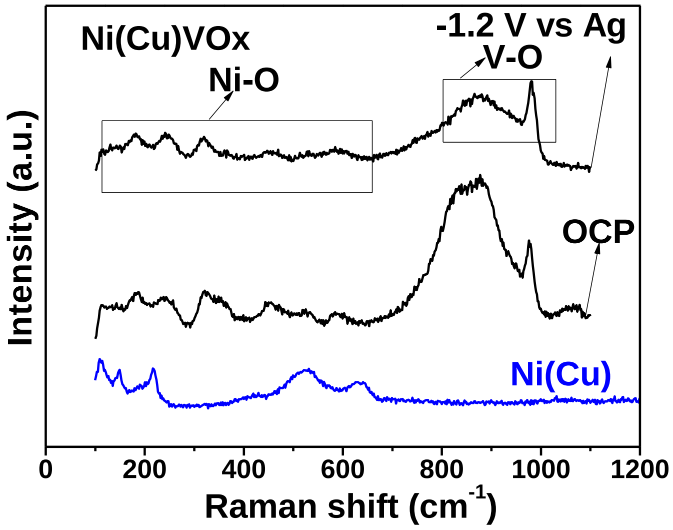

Supplementary Fig. 33 Semi in-situ Raman Spectroscopy. Raman spectroscopy for Ni(Cu)VOx collected at open circuit potential (OCP) and semi in-situ condition after applying a –1.2 V (*vs*. Ag) for 20 min and the Ni(Cu) electrode under OCP.

Supplementary Tables

Supplementary Table 1 Parameters used in the XAS fits of metallic Ni.

**Fit 1:** Fit with *fcc* Ni: E0 = 8337.7 eV, S02 = 0.817, R = 0.0016, Reduced Chi Square = 492				
**Distance**	**Number**	**Distance (structure)**	**Distance (fit)**	σ^2
Ni-Ni	12	2.49	2.48	0.0060
Ni-Ni	6	3.52	3.51	0.0085
Ni-Ni	24	4.31	4.32	0.0089
Ni-Ni-Ni	48	3.73	3.68	0.0107
Ni-Ni-Ni	120	4.64	4.55	0.0039
Ni-Ni-Ni	36	4.97	5.05	0.0138
**Fit 2:** Fit to Ni(Cu) with parameters of Fit 1 (floated): E0 = 8341.42 eV, S02 = 0.625, R = 0.025, Reduced Chi-square = 492				
**Distance**	**Number**	**Distance (structure)**	**Distance (fit)**	σ^2
Ni-Ni	12	2.49	2.51	0.0065
Ni-Ni	6	3.52	3.57	0.0042
Ni-Ni	24	4.31	4.32	0.0069
Ni-Ni-Ni	48	3.73	3.68	-0.0257
Ni-Ni-Ni	120	4.64	4.61	-0.0003

Ni-Ni-Ni	36	4.97	5.09	0.0136

Fit 3: Fit to Ni(Cu) with parameters from a combination of metallic Ni and DFT optimised Ni(Cu). An improved fit to the EXAFS. E0 = 8341.42 eV, S02 = 0.625, R = 0.025, Reduced Chi-square = 492

**Distance**	**Number**	**Distance (structure)**	**Distance (fit)**	$\boldsymbol{\sigma^2}$
Ni-Ni	12	2.49	2.52	0.0053
Ni-Ni	6	3.52	3.58	0.0021
Ni-Ni	24	4.31	4.35	0.01159
Ni-Ni-Ni	36	4.97	5.12	0.01159
Ni-Cu-Ni	18	4.31	4.52	0.00104

Fit 4: Fit to $NiVO_x$ with Ni parameters. S02 = 0.362; E0 = 8341.42 eV, R = 0.111, Reduced Chi-square = 771.8

**Distance**	**Number**	**Distance (structure)**	**Distance (fit)**	$\boldsymbol{\sigma^2}$
Ni-Ni	12	2.49	2.48	0.0067
Ni-Ni	6	3.52	3.34	0.0028
Ni-Ni	24	4.31	4.31	0.0040
Ni-Ni-Ni	48	3.73	3.84	-0.016
Ni-Ni-Ni	120	4.64	4.61	-0.001
Ni-Ni-Ni	36	4.97	5.01	0.0122

**Fit 5:** Fit to NiVOx with a combination of metallic Ni and Ni oxide (improved fit). E0 = 8341.96 eV, S02 = 0.365, R = 0.0485, Reduced Chi-square = 196.6				
**Distance**	**Number**	**Distance (structure)**	**Distance (fit)**	σ^2
Ni-Ni	12	2.49	2.48	0.0067
Ni-Ni	6	3.52	3.34	0.0103
Ni-Ni	24	4.31	4.31	0.0105
Ni-Ni-Ni	120	4.64	4.61	0.0027
Ni-Ni-Ni	36	4.97	5.01	0.0157
NiO	3	2.08	2.02	0.001
Ni-Ni (NiO)	3	2.95	3.00	0.004

**Fit 6:** Fit to Ni(Cu)VOx using Ni parameters from Fit 1. E0 = 8336.9 eV, S02 = 0.521, R = 0.0016, Reduced Chi-square = 492				
**Distance**	**Number**	**Distance (structure)**	**Distance (fit)**	σ^2
Ni-Ni	12	2.49	2.49	0.006
Ni-Ni	6	3.52	3.50	0.0121
Ni-Ni	24	4.31	4.30	0.0096
Ni-Ni-Ni	48	3.73	3.78	0.052
Ni-Ni-Ni	120	4.64	4.64	0.0051

Ni-Ni-Ni	36	4.97	4.97	0.0159

Fit 7: Fit to Ni(Cu)VO_x using a combination of Ni parameters from Fit 1 and nickel oxide parameters. E0 = 8337.7 eV, S02 = 0.774, R = 0.0016, Reduced Chi-square = 492

**Distance**	**Number**	**Distance (structure)**	**Distance (fit)**	**σ^2**
Ni-Ni	12	2.49	2.48	0.006
Ni-Ni	6	3.52	3.52	0.0121
Ni-Ni	24	4.31	4.30	0.0096
Ni-Ni-Ni	48	3.73	3.73	0.065
Ni-Ni-Ni	120	4.64	4.55	0.0215
Ni-Ni-Ni	36	4.97	504	0.0174
Ni-O	1.0	2.08	2.05	0.0053

Supplementary Table 2 Parameters used in the XAS fits of V.

Fit to NiVOx at the V-edge. E0 = 5468 eV, S02 = 0.527, R = 0.0109, Reduced Chi-square = 35.64				
**Distance**	**Number**	**Distance (structure)**	**Distance (fit)**	**σ^2**
V-O1	2	N/A	1.65	0.006
V-O2	2	N/A	1.78	0.0121
V-Ni/V	1	N/A	3.08	0.0096
Fit to Ni(Cu)VOx at the V-edge. E0 = 5468 eV, S02 = 0.512, R = 0.0284, Reduced Chi-square = 35.64				
**Distance**	**Number**	**Distance (structure)**	**Distance (fit)**	σ^2
V-O1	3	N/A	1.81	0.00196
V-O2	2	N/A	2.05	0.00502

Supplementary Table 3 Comparison of HER catalytic activity with reported HER catalysts from non-precious materials in 1 M KOH.

Catalyst	Overpotential in 1 M KOH (*i*R corrected)		Substrate	Medium	Reference
	**10 mA cm^{-2}**	**100 mA cm^{-2}**			
Ni(Cu)VOx	10 mV	42 mV	NF	1 M KOH	this work
NiO/Ni-CNT	85 mV	100 mV	NF	1 M KOH	1
Ni-Co alloy	107 mV	198	Cu foil	1 M KOH	2
Co_3Mo	68	200 mV	NF	1 M KOH	3
Mo doped Ni_2P	78 mV	N/A	NF	1 M KOH	4
h-NiSx	60 mV	~175 mV	NF	1 M KOH	5
NiSe nanowire	96 mV	N/A	NF	1 M KOH	6
Ni_3S_2	170 mV	N/A	NF	1 M KOH	7
NiFe-LDH	210 mV	N/A	NF	1 M KOH	8
$MoNi_4/MoO_2$ @Ni	15 mV	45 mV	NF	1 M KOH	9
Ni–Mn_3O_4	91 mV	N/A	NF	1 M KOH	10

Supplementary Notes

Supplementary Note 1 The reduction behavior of NH_4VO_3.

To see if the NH_4VO_3 precursor was reduced to metallic V, we obtained the XRD spectra of the electrodeposited pure VOx on both FTO and carbon fiber paper. As seen from Supplementary Fig. 5, there are no new peaks that can be assigned to metallic V. This result is in consistence with the XPS and XAS data, where only oxidized V is detected. In addition, previous reports show that metallic V can be obtained *via* electro-reduction of sodium metavanadate in molten salt at high temperatures[11] (> 1,000 K), while room temperature electrochemical reduction can only achieve oxidized vanadium state[12].

Supplementary Note 2 Faradaic efficiency testing.

The plot of the theoretical and experimental amounts of H_2 against time is shown below. The electrolysis was carried out in a two compartment gastight H-cell at a potential of –1.2 V (*vs*. SCE) and the generated H_2 is detected online by a gas chromatograph system at the electrolysis time of 8 min, 16 min and 24 min, respectively (Supplementary Fig. 16). The Faradaic efficiency (FE) was calculated to be 99.5 ± 1% by using the following equation.

$$\mathrm{FE} = \frac{2\mathrm{FV}\upsilon \mathrm{p_0}}{\mathrm{RT_0 I}} \times 100\% \quad \text{Supplementary Equation 1}$$

$$\mathrm{FE} = \frac{2\times 96{,}485\times \mathrm{V}\upsilon\times 1.01\times 10^5}{8.314\times 298.15\times \mathrm{I}} \times 100\% = \frac{0.315\times \mathrm{V}\times\upsilon}{\mathrm{I}} \times 100\% \quad \text{Supplementary Equation 2}$$

FE = Faradaic efficiency; υ = volume concentration of H_2 in the exhaust gas from the cell; V = Ar flow rate (20.0 mL min^{-1}).

The test for FE was carried out on an Autolab potentiostat (Multi Autolab M204) in a

two-compartment gastight H-cell separated by a Nafion 117 membrane (Fuel Cell Store). The working electrode and saturated calomel reference electrode (SCE) were placed in the sealed cathodic compartment. A Pt foil was placed in the anodic compartment as the counter electrode. Each chamber contained 30 mL of 1 M KOH aqueous electrolyte. Prior to HER, Ar (99.995%) was purged into the sealed cathodic electrolyte at a flow rate of 20.0 mL min^{-1} controlled by a mass flow controller (Cole-Parmer) for 30 min. During chronoamperometric electrolysis, Ar gas was continuously bubbled into the cathodic compartment and vented directly into the gas sampling loop (1 mL) of a GC (SHIMADZU GC-2010PLUS) equipped with a packed MolSieve 5A column, a ShinCarbon ST Micropacked column and a packed PoraPLOT Q column. The generated H_2 were analyzed by a thermal conductivity detector (TCD).

Supplementary Note 3 Electrochemically active surface area (ECSA).

The calculation of ECSA is based on the measured double-layer capacitance (C_{dl}) of the synthesized electrodes in 1 M KOH. Briefly, a potential range where no apparent Faradaic process happened was determined first using the static CVs. The charging current i_c, which equals the product of the scan rate (v) and the electrochemical double-layer capacitance (C_{dl}), was measured from the CVs (Supplementary Fig. 17) at different scan rates and follows the equation $i_c = vC_{dl}$. Thus, the ECSA can be calculated with C_{dl} and a known C_s = 0.04 mF cm^{-2} in 1 M KOH based on typical reported values.

Supplementary Note 4 BET measurement.

Apart from ECSA, we also measured the BET specific surface area of Ni, Ni(Cu), NiVOx and Ni(Cu)VOx samples with 17.988, 26.614, 18.994 and 34.232 m^2 g^{-1}, respectively (Supplementary Fig. 20). As shown, the Ni(Cu)VOx still shows the largest specific surface area. Thus, the two techniques show the same trend in surface area. A difference in the measured value between these two techniques is expected due to different working mechanisms, where BET area measures the physiosorption of N_2 through the Van der Waals force in gas phase, while ECSA measures double-layer capacitance between the electrode and electrolyte.

Supplementary Note 5 V K-edge XANES and XPS.

As seen from Supplementary Fig. 21a, the direct interaction between Ni and VOx is clearly seen. In the XAS pre-edge, the effective nuclear charge of the Ni(Cu)VOx and NiVOx is observed at lower energy relative to VOx. In addition, we have also collected the XPS data for the pure VOx, Ni(Cu)VOx and NiVOx. As seen from Supplementary Fig. 21b, compared with Ni(Cu)VOx and NiVOx, the characteristic peak of pure VOx sample located at a higher binding energy position, which not only indicate it has a higher valence state, but further means there is a direct interaction between Ni and VOx.

Supplementary Note 6 Expanded details of the EXAFS fits.

Fits to metallic Ni and Cu-doped Ni (named as Ni(Cu)). The EXAFS data at the Ni K-edge of all four materials give EXAFS that is dominated by the *fcc* structure of metallic Ni. This is clear from the Fourier Transforms of the EXAFS, which can be well fit from the main peaks of metallic Ni, 12 × Ni-Ni @ 2.49 Å; 6 × Ni-Ni @ 3.52

Å; 24 × Ni-Ni @ 4.31 Å; 48 × Ni-Ni-Ni @ 3.73 Å; 120 × Ni-Ni-Ni @ 4.64 Å and 36 × Ni-Ni-Ni @ 4.97 Å. The EXAFS of metallic Ni is well fit with the known *fcc* structure, as shown in Fit 1 (Supplementary Fig. 22). Introducing Cu to Ni lattice affects the long-range order of the Ni-Ni-Ni interactions, which can be seen from the EXAFS of Ni(Cu) with the same parameters as metallic Ni (Fit 2). In this fit, while the inner sphere (*i.e.* the short Ni-Ni distances) is well fit, the longer Ni-Ni-Ni interactions are poorly fit. This is indicated by the Debye Waller parameters (σ^2) going negative when the fit parameters optimized for a Ni lattice are used. This indicates that the Cu is disrupting the order of the Ni lattice. A change to the fitting parameters to include distances calculated by DFT provides more physically sensible XAS fit, as shown in Fit 3.

The interaction of VO_x with metallic Ni and Ni(Cu). To understand the introduction of VO_x into a metallic Ni lattice, as a starting point, the $NiVO_x$ material was fit with the parameters associated with metallic Ni and the result is given in Fit 4 (Supplementary Fig. 22). The XAS could be reasonably well described with the parameters from metallic Ni. However, the EXAFS needed to be dampened substantially compared to the metals collected in same way. (S02 = 0.4 vs. S02 = 0.67). This indicates a secondary effect causing dampening of the EXAFS, potentially disorder caused by lattice distortions as shown in Supplementary Fig. 23 or other effect of the electrode or matrix. Even with the dampened EXAFS, there were still key differences in the long range Ni-Ni-Ni vectors which were not well fit from the basic parameters of metallic Ni. To investigate the nature of the material further, a

secondary contribution from a Ni-O was considered. A contribution like this would be consistent with a material that has Ni-O-VO_x type interactions. The fit improved substantially with the introduction of a small contribution from NiO, as seen in Fit 5 (Supplementary Fig. 22). This indicates the presence of either a secondary phase (for which there was no evidence from XRD or XPS) or a potential interface between VO_x and Ni. The EXAFS for Ni(Cu)VOx could be well fit with two V-O contributions. Adding a second coordination sphere, say a V-Ni distance at ~3 Å, did not improve the fit. This is in consistent with a very disordered 2nd sphere and many slightly different VOx sites throughout the material.

It can be seen from XPS (Supplementary Fig. 7) that there are abundant Ni^{2+} in Ni(Cu)VOx, while no distinct first-shell of Ni-O is observed in Ni K-edge FT-EXAFS (Fig. 3e in manuscript). This is due to that XPS is a surface technique and that XAS in the configuration used for these experiments is a bulk technique. However, in the EXAFS fits of both Ni(Cu)VOx and NiVOx, NiO contributions were considered. This is given in the Supplementary Fig. 22 in Fits 4 and 5 for NiVOx and Fits 6 and 7 for Ni(Cu)VOx "with" and "without" NiO. A small contribution of NiO is indeed found to improve fit to the EXAFS data of NiVOx. The difference between *fcc* Ni and the inclusion of NiO parameters is shown by a comparison of Fit 4 and Fit 5. The improvements in the refinement are present, as indicated by the decrease in the Chi-squared value. The Ni(Cu)VOx was also fit in the same way. In this case, the fit with NiO was close to the fit without NiO. This indicates that the presence or absence of NiO in Ni(Cu)VOx is not above experimental error, but it does not mean it is not

there. As can be seen from Fig. 3a in the Ni K-edge in the manuscript, the white line intensity and its position indicate Ni in Ni(Cu)VOx was also mildly oxidized.

Supplementary Note 7 Fit to the EXAFS taken at the V K-edge of Ni(Cu)VOx and NiVOx.

As V forms only a small amount of the material, it is a very sensitive probe of its local environment. EXAFS taken at the V edge of Ni(Cu)VOx and NiVOx were both very weak compared to that taken on metallic materials. This is clear from the lower intensity of the Fourier Transform (Supplementary Fig. 24 and Supplementary Table 2). Weak EXAFS is often associated with disordered materials. In this case, the likely explanation is that the structure the VOx takes on a number of slightly different but related geometries. Both the XAS of the Ni(Cu)VOx and NiVOx were well with V-O distances. Attempts were made to fit direct Ni-V bonds. However, they could not be fit this way, indicating families of interactions where the VOx is likely bridged to the nickel by an oxygen group, a likely Ni-O-VOx interaction would be consistent with the data taken at the Ni K-edge.

Note that the noise level of the EXAFS at the V edge is not sufficient to unambiguously identify a V-O-Ni interaction. It is consistent with it but it does not unambiguously show it. This interaction is clearer from the analysis of the effectively nuclear charge changes as we describe in detail in the manuscript. Errors on fitting refinements are given in the Supplementary Table 1. In terms of V K-edge, these give an error of ± 0.15 Å on the inner sphere distances.

Supplementary Methods

Preparation of VOx electrode. The preparing of VOx electrode is the same as that of Ni(Cu)VOx. In a typical synthesis, all other conditions are unchanged but with the electrolyte of 7 mM NH_4VO_3 and 0.5 M H_3BO_3 by dissolving the chemicals in 50 mL Milli-Q water under sonication. The electrodeposition was carried out on CHI 760D electrochemical workstation at –2.0 V (vs SCE) for 600 s at room temperature. From Supplementary Fig. 1, the NH_4VO_3 reduction peak appears at –2.09 V (vs. SCE) and the reverse potential direction scan produces a current loop at –2.02 V and –1.76 V indicative of a nucleation and growth mechanism for VOx deposition.

Preparation of NiVOx electrode. The preparing of NiVOx electrode is described as below. The electrolyte contains 0.5 M $NiSO_4$, 7 mM NH_4VO_3 and 0.5 M H_3BO_3 by dissolving each chemical in 50 mL Milli-Q water under sonication. The electrodeposition was carried out on CHI 760D workstation at –2.0 V (vs SCE) for 600 s at room temperature.

Preparation of Ni and Ni(Cu) electrodes. For Ni deposition, the electrolyte contains 0.5 M $NiSO_4$ and 0.5 M H_3BO_3. For Ni(Cu) deposition, the electrolyte contains 0.5 M $NiSO_4$, 12.5 mM $CuSO_4$ and 0.5 M H_3BO_3, by dissolving each chemicals in 50 mL Milli-Q water under sonication. The electrodeposition was carried out on CHI 760D electrochemical workstation at –2.0 V (vs SCE) for 600 s at room temperature.

X-ray diffraction spectroscopy (XRD). XRD measurements were performed with PANalytical X'Pert Empyrean instrument equipped with standard Cu anode, K-α wavelength = 1.54 Å. The typical scan range (2θ) was 20° to 80°, collected with step

size of 0.039^{o} s^{-1}. For physical characterization purpose, fluorine-doped tin oxide (FTO) glass was used as the substrate to eliminate the influence of Ni signal from NF substrate.

Scanning electron microscopy and transmission electron microscopy. Scanning electron microscopy (SEM, JSM-7001F), transmission electron microscopy (TEM), high-resolution transmission electron microscopy (HR-TEM), and energy-dispersive X-ray spectroscopy (EDS) mapping images were obtained with a JEOL-F200 instrument. To prepare the TEM sample, a piece of NF with the deposited catalyst was sonicated from the NF substrate in an ethanol solution. The resulting top solution was then drop-casted onto Cu-grid and dried in room temperature.

X-ray photoelectron spectroscopy. Chemical compositions of the samples were analyzed by X-ray photoelectron spectroscopy (XPS, Thermo ESCALAB250i X-ray photoelectron spectrometer). The binding energies reported in this study were calibrated to adventitious hydrocarbon at 284.7 eV.

Supplementary references

1. Gong, M. et al. Nanoscale nickel oxide/nickel heterostructures for active hydrogen evolution electrocatalysis. *Nat. Commun.* **5**, 4695 (2014).

2. Darbanda, G., Aliofkhazraeia, M., Rouhaghdama, A., Kian. M.A. Three-dimensional Ni-Co alloy hierarchical nanostructure as efficient non-noble-metal electrocatalyst for hydrogen evolution reaction. *Appl. Surf. Sci.* **465**, 846 (2019).

3. Chen, J.Y. et al. Nesting Co_3Mo binary alloy manoparticles onto molybdenum oxide nanosheet arrays for superior hydrogen evolution reaction. *ACS Appl. Mater. Interfaces*. **11**, 9002 (2019).

4. Sun, Y. et al. Mo doped Ni_2P nanowire arrays: an efficient electrocatalyst for the hydrogen evolution reaction with enhanced activity at all pH values. *Nanoscale*. **9** 16674 (2017).

5. You, B., Sun, Y. Hierarchically porous nickel sulfide multifunctional superstructures. *Adv. Energy Mater*. **6**, 1502333 (2016).

6. Tang, C., Cheng, N., Pu, Z., Xing W., Sun X. NiSe nanowire film supported on nickel foam: an efficient and stable 3D bifunctional electrode for full water splitting. *Angew. Chem. Int. Ed.* **54**, 9351-9355 (2015).

7. Feng, L.L. et al. High-index faceted Ni_3S_2 nanosheet arrays as highly active and ultrastable electrocatalysts for water splitting *J. Am. Chem. Soc.* **137**, 14023-14026 (2015).

8. Luo, J. et al. Water photolysis at 12.3% efficiency via perovskite photovoltaics and Earth-abundant catalysts. *Science*. **345**, 1593-1596 (2014).

9. Zhang, J. et al. Efficient hydrogen production on $MoNi_4$ electrocatalysts with fast water dissociation kinetics *Nat. Commun.* **8**, 15437 (2017).

10. Li, X. et al. Enhancing alkaline hydrogen evolution reaction activity through Ni–Mn_3O_4 nanocomposites. *Chem. Commun*. **52**, 10566-10569 (2016).

11. Weng W. et al. One-step electrochemical preparation of metallic vanadium from sodium metavanadate in molten chlorides. *Int. J. Refract. Met. H.* **55**, 47-53 (2016)

12. Liu, B. et al. The redox behavior of vanadium in alkaline solutions by cyclic voltammetry method. *Electrochimi. Acta*. **76**, 262-269 (2012).
