# Supplementary material for: Implanting Ni-O-VOx sites into Cu-doped Ni for low-overpotential alkaline hydrogen evolution
Source: Nat Commun. 2020 Jun 1;11:2720. doi: 10.1038/s41467-020-16554-5 (PMC7264301; doi:10.1038/s41467-020-16554-5)
Supplement: Supplementary file 2 — Peer Review File [file 41467_2020_16554_MOESM2_ESM.docx]

Peer Review Information

**Journal:** Nature Communications

**Manuscript title:** Click here to enter text.

**Corresponding author name(s):** Click here to enter text.

**Editorial notes:**

**Transferred manuscripts**

This manuscript has been previously reviewed at another journal that is not operating a transparent peer review scheme. This document only contains reviewer comments and rebuttal letters for versions considered at *Nature Communications*.

**Transferred manuscripts (no peer review at *Nature Communications*)**

This manuscript has been previously reviewed at another journal that is not operating a transparent peer review scheme. The manuscript was considered suitable for publication without further review at *Nature Communications*.

**Redactions – transferred manuscripts (mention of previous referee reports from elsewhere)**

This manuscript has been previously reviewed at another journal that is not operating a transparent peer review scheme. This document only contains reviewer comments and rebuttal letters for versions considered at *Nature Communications*. Mentions of prior referee reports have been redacted.

**Redactions – transferred manuscripts (mention of the other journal)**

This manuscript has been previously reviewed at another journal that is not operating a transparent peer review scheme. This document only contains reviewer comments and rebuttal letters for versions considered at *Nature Communications*. Mentions of the other journal have been redacted.

**Redactions – unpublished data**

Parts of this Peer Review File have been redacted as indicated to maintain the confidentiality of unpublished data.

**Redactions – confidential patient information**

Parts of this Peer Review File have been redacted as indicated to maintain patient confidentiality.

**Redactions – published data**

Parts of this Peer Review File have been redacted as indicated to remove third-party material where no permission to publish could be obtained.

**Amendments – published data**

[Figure X/The figure on page X] in this Peer Review File has been amended to remove third-party material where no permission to publish could be obtained.

**Published data (credit line for image)**

[Figure X/The figure on page X] in this Peer Review File is reproduced with permission from [Copyright Holder].

**Redactions – reviewer opt-out**

Parts of this Peer Review File have been redacted as indicated as we could not obtain permission to publish the reports of reviewer ###.

**Reviewer comments in marked-up manuscript**

In their review of the [first/second/third/...] version of this manuscript, reviewer ### added their comments to the manuscript file. These comments, excluding minor textual revisions, have been copied into this Peer Review File.

**Reviewer comments & decisions:**

**Reviewer comments, first version:**

Reviewers' comments:

Reviewer #1 (Remarks to the Author):

This paper reports the characterisation of the structural and reactive properties of Cu-doped Ni catalyst implanted on
active Ni-O-VOx sites ( Ni(Cu)VOx) for alkaline water electrolysis.
The presence of VOx can cause important modification to the Ni lattice
creating an enlarged electrochemically active surface area,
which provides the Ni(Cu)VOx electrode with extremely high HER activity and long-term durability.
The paper points to new interesting results, it is well written and of interest for the Nat Comm community.
I have a couple of comments on the first principles calculations which should be addressed.
1) As first I would like to ask the authors to provide some comments on the accuracy of the used method (DFT with PBE functional)
for the calculations. In particular how accurate is to describe the radical state? Do you observe any localization/delocalization error?
2) The calculations are only done in the gas phase. How accurate is this approximation to describe the actual operative conditions?
what would be the influence of the solvent? How would the calculated properties change if solvent is added to the different systems?


Reviewer #3 (Remarks to the Author):

This paper reports on tuned nickel-based catalysts as electrocatalysts for hydrogen evolution reaction (HER) for alkaline water electrolysis. Ni, Cu and VOx are formed on the surface of nickel foam by electrodeposition method, and VOx plays an important role in excellent HER activity. The authors also suggest the causes of activity and durability through various characterizations and DFT calculations.

Ni (Cu) VOx was formed on two supports, Nickel foam (NF) and FTO. The authors explain the use of the FTO for physical characterization purposes. This approach is understandable, but the reviewer believes that it is necessary to confirm that the active metals loaded on two different supports are in the same state. Several methods may be considered, such as XPS (Depth profiling) or ICP analysis of surface scraped powder.
Considering the standard reduction potential of Ni, Cu, V or VOx, at -2.0 V (vs. SCE), all elements are expected to be electrodeposited simultaneously. In this case, the composition of each layer (by thickness) may be different due to the difference in the reduction properties of each metal. However, in this paper, the analysis of the structure of the active metal is not clear. It is also likely that at significantly lower potentials, such as -2.0 V, vanadium precursors may be reduced to V (s) as well as VOx. The authors confirm all existing forms as VOx and continue discussion.
Further explanation is needed for the expressions Cu2 + / Ni2 + = 2.5% and Cu / Ni = 11.4%. The ratio expression and the "%" unit are used together, so the meaning is not clear. In order to compare HER electrochemical activity, it is important to know the Cu / Ni and V / Ni ratio information together with the absolute amount of active metal loaded in NF. Although the text says "Catalyst mass loading on NF is: (3.0 +/- 0.2) mg / cm ^ 2", it is thought that the ratio of each metal and the "absolute amount of metal" should be provided together.
After electrodepostion for 600 seconds, galvanic displacement reactions of “vanadium and / or nickel metals” and Cu can occur if Cu ions remain in the precursor solution and are contained in the electrolyte for a short time under OCV conditions. In this case, the surface of the active metal is probably rich in Cu. These experimental variables can make a real difference in the structure of the metal and lead to different results than the basic structure used in the DFT. In spite of the results of Fig. 3 (a), the authors do not consider the possibility of existing nickel in NiOx form in the DFT.
The authors argue that VOx plays a major role in HER response activity and durability. In 'parameter fitting' and 'structure for DFT calculation', the position and role of VOx are not clear. In terms of durability, only the dissolution of "physically absorbed and weakly bonded VOx" is mentioned. Even after AST, large amounts of V are still observed. Further analysis of the location and role of V (s) or VOx seems necessary.
Information about the Electrolyte used in the Electrodeposition process needs to be added.
ECSA calculation results can be correlated with BET specific surface area results.
It would be nice to express that Fig. 2 (e) is vanadium. In addition, Ni and Cu information as well as vanadium may be helpful in comparing durability.
It appears that "Ar gas" and "N2 flow rate" are used simultaneously in "3.6 Supplementary".

In summary,
It is clear that the Ni-based Non-PGM catalyst presented in this paper shows very good HER activity. The authors also attempted to suggest the structure of the catalyst and the reason for its high activity through XAS analysis and DFT. However, there is still some ambiguity in the structure and discussion. After some key updates, the reviewer suggests that this article may be considered for publication in this or other journals.

**Author rebuttal, first version:**

This paper reports the characterisation of the structural and reactive properties of Cu-doped Ni catalyst implanted on active Ni-O-VOx sites (Ni(Cu)VOx) for alkaline water electrolysis. The presence of VOx can cause important modification to the Ni lattice creating an enlarged electrochemically active surface area, which provides the Ni(Cu)VOx electrode with extremely high HER activity and long-term durability. The paper points to new interesting results, it is well written and of interest for the Nat Comm community.

I have a couple of comments on the first principles calculations which should be addressed.

(1) At first I would like to ask the authors to provide some comments on the accuracy of the used method (DFT with PBE functional) for the calculations. In particular how accurate is to describe the radical state? Do you observe any localization/delocalization error?

**Response:** Many thanks for the reviewer’s comments. Previous studies have shown DFT with PBE functional is a reliable and effective method to study the catalytic activity and reaction mechanism of HER and other reactions, such as OER, ORR and so on (*Nat. Commun.* **7**, 10990 (2016), *Nat. Commun.* **10**, 1348 (2019), *Nat. Energy* **2**, 17111 (2017)). In particular, the free energy of the radical state of HER, i.e. H*, can be well descripted by DFT with PBE functional (e.g. *Nat. Commun.* **7**, 10990 (2016), *Nat. Commun.* **10***,* 1348 (2019), *Nat. Commun.* **8**, 14969 (2017), *J. Am. Chem. Soc.* **139**, 12370 (2017)). It is established that the lack of derivative discontinuity of the functional with respect to the number of electrons and the self-interaction error in local/semi-local functionals can lead to stabilization of electron delocalization and significantly underestimates the band gap of the materials. Hubbard U or hybrid functionals, such as PBE0 and HSE06, are among the commonly applied methods to overcome this problem, which however have their shortcomings. The Hubbard U parameter can be seen as an empirical parameter, where a correct and unambiguous value can be hard to obtain or justify. HSE06 also has empirical parameters in the form of the Hartree-Fock fraction or the range separation. In addition, both are computationally very expensive to employ for determining reaction mechanisms, where a large number of calculations are typically needed. In comparison, PBE functional has been shown to be quite good in capturing structural properties of both bulk phase and the surface of the materials. While a certain degree of charge delocalization is inevitable, the effect is not significant in our case, as we are dealing here with a metallic system. In fact, the use of hybrid functionals can be more erroneous for metallic systems as has been argued before. In line with previous calculations, we believe that the charges and adsorbed intermediates are adequately described at the GGA-PBE level of theory for the type of material used here.

(2) The calculations are only done in the gas phase. How accurate is this approximation to describe the actual operative conditions? What would be the influence of the solvent? How would the calculated properties change if solvent is added to the different systems?

**Response:** Thanks the reviewer’s comments. In our calculations, we used the electrochemical framework developed by Nørskov and his co-workers (*J. Electrochem. Soc.* **152**, J23-J26 (2005)), which are popularly used for calculation of the catalytic activity and mechanism study of the electrocatalytic HER and other reactions, as we mentioned above. Although our calculations are done in the gas phase, similar to Nørskov’s approach, we believe our calculation results are accurate in describing realistic operating conditions because the solvation effect is very small for HER and can be safely ignored, as well explained and confirmed in many previous studies (e.g. *J. Electrochem. Soc.* **152**, J23-J26 (2005), *Nat. Mater.* **15**, 48-53 (2016), *Nat. Commun.* **7**, 10990 (2016)). Therefore, it is concluded that the solvent effects don’t cause appreciable change to activities for the systems reported here, compared to that in vacuum.

Relevant discussion is added into the revised manuscript in “DFT calculations” in “Methods” section.

**Response to Reviewer: #2**

This manuscript reports a facilely prepared Ni-based electrocatalyst, Ni(Cu)VOx, which display a very high activity and superior stability for HER in 1 M KOH. Comparative electrochemical studies of Ni, Ni(Cu), NiVOx, and Ni(Cu)VOx demonstrated that implanting of VOx to the Cu-doped Ni catalyst significantly enhanced the catalytic activity and stability for HER. The Ni(Cu)VOx catalyst was characterized by XRD, SEM, EDX, TEM, XPS, and ICP. Moreover, the geometric and electronic structures of the as-prepared catalysts were studied by XANES and EXAFS. The DFT calculations of the active surface were performed, providing reasonable explanation for the origin of the high activity. In view of the hot topic and the impressive results of this part of work, the manuscript may suitable for publication in Nature Communications after the following problems have been addressed.

**Response:** Many thanks to the reviewer’s insightful comments. After receiving the reviewer’s comments, we have carried out a series of new experiments and DFT computations in order to provide further evidence to elucidate the structure of the materials and to support the reaction mechanism. We believe we have shifted our work to the next level of proof and quality and gained a deeper understanding of our system.

We have provided point-by-point responses to the reviewer’s comments and also revised the manuscript and Supplementary Information and highlighted yellow.

(1) In Abstract, “…exhibits a zero overpotential for HER”, more accurate expression should be “…exhibits a zero onset overpotential for HER”.

**Response:** The expression has been changed accordingly. Thanks for the suggestion.

(2) The XPS spectra of Ni(Cu) and NiVOx catalysts and the molar ratios of Cu/Ni for Ni(Cu) and V/Ni for NiVOx analyzed by ICP should be given in the Supporting Information (SI). Comparison of XPS spectra of Ni(Cu), NiVOx, and Ni(Cu)VOx may give some valuable information on the changes in the oxidation state of Ni on the surface of the as-prepared catalysts.

**Response:** Thanks for the reviewer’s suggestions. We have carried additional XPS and ICP for Ni(Cu) and NiVOx, and the results are shown in Fig. R1 below. For Ni(Cu), the Ni2p and Cu2p XPS shows both the metallic Ni and Cu, as well as slightly oxidized Ni and Cu, which are similar to Ni(Cu)VOx. For NiVOx, a higher oxidation degree of Ni was observed, which is in consistent with the XAS data. In addition, the molar ratio of Cu : Ni = 0.168 for Ni(Cu) and V : Ni = 0.036 for NiVOx have been determined by ICP-OES. These values are slightly higher than that in Ni(Cu)VOx, suggesting a small change in composition of Ni, Cu and V elements as a result of the electrodeposition process.

Relevant discussion is added into the “Synthesis and characterization of the Ni(Cu)VOx catalyst, Evaluation of electrocatalytic activity and stability” in the revised manuscript and Supplementary Fig. S8 in the Supplementary Information.

**Fig. R1.** (a, b) Ni2p and Cu2p XPS spectra of Ni(Cu) and (c, d) Ni2p and V2p for NiVOx.

(3) It is better to change the y-coordinate from “Concentration/mg/L” to “[VOx]/mg L^−1^”, which is shorter and clearer. From the y-coordinate of “Concentration/mg/L and the caption for Fig. 2e, one cannot clearly understand what the authors want to express.

**Response:** As suggested by the reviewer, the y-coordinate in Fig. 2e has been changed from “Concentration/mg/L” to “[vanadium] / mg L^-1^”.

(4) The method used for iR compensation should be briefly described.

**Response:** Thanks the reviewer’s suggestion. The 95 % *iR* compensation was carried out automatically by the potentiostat using its standard *iR* compensation function. The way this is done has been defined in a publication by Peixin He and Larry R. Faulkner (*Anal. Chem.* **58**, 517-523 (1986)), and also quoted as the following:

“The system works about a test potential at which no Faradaic reaction occurs. By examining the current response to a **Δ***E* = 50 mV step across this test point, the instrument first determines the amount of uncompensated resistance. Signal-averaged currents at 54 and 72 *µ*s after the step edge are extrapolated backward to provide a zero-time current, which is **Δ***E/R_u_*. The system then stages the application of positive feedback by using a multiplying digital-to-analog converter as a digitally controlled potentiometer. A test for potentiostatic stability is made after each stage. This test is carried out by applying a 50-mV step across the test potential and watching for ringing in the output of the *I/V* converter. When this ringing reaches a defined threshold, the system attempts to stabilize the network capacitively. When full compensation is reached or when the system can take no further action without crossing the allowed ringing threshold, the process ceases”.

The *iR* compensation information and citation are added into the revised manuscript in “Electrochemical measurement” in “Methods” section.

(5) It is better to give the *R*_ct_ data obtained from Fig. 2f for different catalysts in the SI.

**Response:** As suggested by the reviewer, the *R*_ct_ value of each catalyst is added into Fig. 2f with relevant discussion in “Electronic and atomic structure analysis” section in the revised manuscript.

(6) The plot of the theoretical and experimental amounts of H_2_ against time for calculation of Faradaic efficiency should be given in the SI.

**Response:** Thanks the reviewer’s comments. The plot of the theoretical and experimental amounts of H_2_ against time have been added into Supplementary Information and also shown below (Fig. R2). The electrolysis was carried out in a two compartment gastight H-cell at a potential of –1.2 V (*vs*. SCE) and the generated H_2_ is detected online by a gas chromatograph system at the electrolysis time of 8 min, 16 min and 24 min, respectively. The Faradaic efficiency (FE) was calculated to be 99.5 ± 1% by using the following equation.

$$FE=\frac{2FVʋp_{0}}{RT_{0}I}\times100\%$$

$$FE=\frac{2\times96,485\times Vʋ\times1.01\times{10}^{5}}{8.314\times298.15\times I}\times100\%=\frac{0.315\times V\timesʋ}{I}\times100\%$$

FE = Faradaic efficiency; ʋ = volume concentration of H_2_ in the exhaust gas from the cell; V = Ar flow rate (20 mL min^-1^).

**Fig. R2.** Theoretical *vs*. experimental amounts of H_2_ during bulk water electrolysis. The electrolysis was carried out in a two compartment gastight H-cell at a potential of –1.2 V (*vs*. SCE) and the generated H_2_ is detected online by a gas chromatograph system at the electrolysis time of 8 min, 16 min and 24 min, respectively.

The details have been added into the revised Supplementary Information in section “3.6. Supplementary Faradaic efficiency testing”.

(7) At page 13: “… results in weak H-adsorption on Ni(111), i.e. the enhanced HER activity”. Such a description may give neophytes a confused impression that the weaker the H-adsorption is, the higher the activity will be. In fact, an optimal H-adsorption according to Sabatier’s principle (H-absorption close to zero) is required for high activity.

**Response:** Thanks for the reviewer’s comments and suggestions. We have revised the description in the manuscript to avoid the confusion: “results in a closer optimal value of $\left| {\Delta G}_{H^{*}} \right|$ on Ni(111)” in “Theoretical simulations” section.

(8) The descriptions: “vanadium oxide doped Ni(111) (V-Ni(111)) and vanadium oxide and Cu co-doped Ni(111) (V/Cu-Ni(111))” and “To understand the vanadium oxide and Cu co-doping effects” at page 12, “For vanadium oxide and Cu co-doped Ni(111)” and “the V and Cu co-doping generates …” at page 13, and Experimental observations of V and Cu co-doped Ni(111) structure …”, are questionable. In all optimized structural models of Ni(Cu)VOx given in the manuscript and the SI, the VOx is not doped in the lattice of Ni, and it is only implanted on the surface of Ni. It should be clear demonstrated that vanadium oxide is co-doped with Cu into the Ni lattice or only implanted on the surface of Ni catalyst.

**Response:** Thanks the reviewer’s comments. As seen from Fig. 1e-h the TEM EDS mapping in the manuscript and the XPS depth profiling (Fig. R3), it shows that VOx are distributed uniformly in the composite. Thus, in the revised version, we constructed a DFT model of Ni(111) with VO_3_ cluster inside Ni lattice (Fig. R4) to simulate the case that VOx located in Ni lattice. We find that the ${\Delta G}_{H^{*}}$ at site 1 is –0.24 eV, which is less negative than that on bare Ni(111) (–0.32 eV), indicating that VOx located inside Ni lattice can also enhance the HER activity. However, compared to the VOx implanted on Ni(111) surface, their contribution to HER enhancement is very small. It is also noted that ${\Delta G}_{H^{*}}$ at other sites for Ni(111) with VO_3_ cluster inside Ni lattice are comparable or more negative (–0.32 ~ –1.16 eV) than that on bare Ni(111) (–0.32 eV). Therefore, it can be concluded that VOx is uniformly doped into Ni lattice but the surface VOx contributes mostly to the enhanced HER.

**Fig. R3.** XPS depth profile of Ni(Cu)VOx.

**Fig. R4.** (a) The optimized structure of VO_3_ cluster in Ni lattice. (b) The optimized structure of H* adsorbed at site 1 on Ni(111) surface with VO_3_ cluster in Ni lattice. The black numbers in (a) denote the possible adsorption sites for H* closed to the oxidized V cluster VO_3_, and the corresponding ${\Delta G}_{H^{*}}$ are listed in the table on the bottom.

We have added relevant discussion in the revised manuscript in “Theoretical simulations” and section “3.9. Supplementary DFT theoretical simulation (Supplementary Fig. S32) and XPS depth profile (Supplementary Fig. S31)” in Supplementary Information.

(9) It is suggested to remove the paragraph “Finally, we demonstrated ……of Co control sample (Supplementary Fig. S25 & S26)” from the Discussion part to the end of Results part.

**Response:** Thanks the reviewer’s suggestion. The mentioned paragraph and data have been removed from the manuscript and Supplementary Information.

(10) In the manuscript, the only descript for the function of doped Cu is “the value of ${\Delta G}_{H^{*}}$ is further improved to 0 eV because of the weak binding of H* on Cu compared to that on Ni.” Authors repeatedly emphasize that the “superactive Ni-O-VOx sites” are the active sites for HER. In this case, how “the weak binding of H* on Cu” influence the activity of HER?

**Response:** From Fig. 4d in the manuscript, we can see that for the bare Ni(111), the ${\Delta G}_{H^{*}}$ is quite negative (–0.32 eV), which indicate a strong interaction between H^*^ and Ni(111), manifesting poor HER reaction kinetics. Introducing oxidized V cluster VO_4_ on Ni(111) (V-Ni(111)) significantly decrease the value of ${\Delta G}_{H^{*}}$ to –0.14 eV, suggesting an enhanced HER activity compared to bare Ni(111).

Further doping Cu atom into the Ni lattice close to VO_4_ cluster (V/Cu-Ni(111)) can adjust the value of ${\Delta G}_{H^{*}}$ close to 0 eV (the optimum value of $\left| {\Delta G}_{H^{*}} \right|$ for HER), indicating the highest HER activity among those three catalysts. The reason is because on Ni(111) or V-Ni(111) surface the adsorbed H* bounds to three Ni atoms (Fig. 4a and 4b in manuscript), while on V/Cu-Ni(111) surface the adsorbed H* bounds to two Ni atoms and one Cu atom (Fig. 4c in manuscript). As binding energy of H* on Ni is stronger than that on Cu (*J. Electrochem. Soc.* **152**, J23-J26 (2005)), the substitution of Cu on Ni surface can increase the value of ${\Delta G}_{H^{*}}$ close to 0 eV. Their HER activities follows the order in the sequence of V/Cu-Ni(111) > V-Ni(111) > Ni(111). In addition, it is expected the doping of metallic Cu can accelerate electron conductivity and further enhance HER activity, which is also evident from the EIS (Fig. 2f in manuscript) test.

We have added more detailed explanation in the revised manuscript in “Theoretical simulations” and “Electronic and atomic structure analysis” sections.

(11) In the Supplementary Fig. S23, the ${\Delta G}_{H^{*}}$ values for Ni in different positions of the NiVOx model have been given. Similarly, the ${\Delta G}_{H^{*}}$ values for Ni in different positions of the Ni(Cu)VOx model should also be calculated. Comparison of the ${\Delta G}_{H^{*}}$ values for the Ni sites of NiVOx and Ni(Cu)VOx in the same position relative to VOx may provide a better understanding on the influence of doped-Cu on the ${\Delta G}_{H^{*}}$ of Ni.

**Response:** Thanks the reviewer’s comments. Accordingly, we have carried out DFT calculations of the ${\Delta G}_{H^{*}}$ values for Ni in different positions of the Ni(Cu)VOx model, as shown in Fig. R5 and Supplementary Fig. S29. For NiVOx and Ni(Cu)VOx, the active sites are both at site 1, as it has the optimized ${\Delta G}_{H^{*}}$ values. However, doping NiVOx with Cu leads to the ${\Delta G}_{H^{*}}$ value approaching to 0 eV, compared to the ${\Delta G}_{H^{*}}$= –0.14 eV of NiVOx, thus demonstrating the substitution of Cu for Ni can increase the value of ${\Delta G}_{H^{*}}$ close to 0 eV to allow it has the best HER performance, as also explained in our response to question # 10.

**Fig. R5.** Structure of the V/Cu-Ni(111) used in our calculations. The magenta numbers denote the possible adsorption sites for H* closed to the oxidized V cluster VO_4_, and the corresponding ${\Delta G}_{H^{*}}$ are listed in the table on the right.

We have added this discussion into the revised manuscript in “Theoretical simulations” section and “3.9. Supplementary DFT theoretical simulation (Supplementary Fig. S30)” in Supplementary Information.

(12) The statement about the valence state of V with XANES in lines 202-205 is not accurate. In fact, from the adsorption edge positions of Ni(Cu)VOx and NiVOx (Fig. 3c), the oxidation state of V in Ni(Cu)VOx is very close to +4, while the oxidation state of V in NiVOx is close to +5. The varied oxidation states of V, the increased white line intensity in Cu K-edge, and the electronic interaction between V and Cu should be discussed, which may be crucial to the significant improvement of HER performance. The sentence at page 10, “…consistent with the XPS results (Supplementary Fig. S11)”, is not correct. The Supplementary Fig. S11c exhibits the V 2p in Ni(Cu)VOx after HER, not the as-synthesized catalyst. Maybe, the authors want to mention the XPS in Supplementary Fig. S6. Besides, the data of O 1s in Supplementary Fig. S11 was fitted poorly.

**Response:** Thanks the reviewer’s comments. Here we address the reviewer’s comments in two parts.

*Part 1: Using the XANES position of the V K-edge data to assign oxidation state.*

We believe it is better not to interpret oxidation state from the position of the XANES alone. This is because the likely coordination numbers (4 coordinate *vs* 5 coordinate) can also impact the XANES position (*J. Am. Chem. Soc*, **132**, 4006-4015 (2010)), as can the nature of the bonding (*Structure and Bonding*, **142**, 155-184 (2012)), for example OH^-^ *vs* O^2-^. For this reason, in the manuscript, we make a point to not “over interpret” the data and leave the assignment of the V oxidation state of 4+/5+. This is in consistent with the XPS results in Supplementary Fig. S7 in the updated Supplementary Information, as corrected by the reviewer.

The reviewer might ask based on this, why we don’t interpret coordination number from EXAFS. While this is accurate in some systems, we do not believe it is correct to do so in this case, because the inherent disorder in the system can dampen the EXAFS peaks, making it difficult to sort out coordination number from disorder. A simple calculation demonstrating this point is given in Supplementary Fig. S23.

*Part 2: The white line intensity of the Copper.*

We assume the reviewer means the XANES shape at the Cu K-edge, as there are not very substantial intensity differences. It should be mentioned that at the time of submission, we had not been able to collect copper EXAFS on these materials. As also requested by the reviewer in question #16, fortunately, we have a chance to re-take the XAS beamline. We have updated Fig. 3b and the pristine and new data have similar results at XANES.

There are subtle changes in the XANES of copper, which can be caused by many reasons (*Structure and Bonding*, **142**, 155-184 (2012)). The problem for interpretation in this case is there are multiple plausible interpretations, and the identifiable changes are small. The most likely explanation is that there are differences in the structure disorder of the materials that split the degeneracies of the transitions. There are also possible artifacts that may contribute to these changes, particularly self-absorption. The foil reference used in the EXAFS experiments is very thin and the data is taken in transmission mode. The differences seen in Fig. 3b are also plausibly due to spectroscopic artifacts of self absorption.

Further, in terms of the interaction effect between Cu and V on the HER performance, we have prepared the Cu and CuVOx control electrodes. The HER performances were measured under the same conditions and it shows that both electrodes display similar HER activity (Fig. R6). Of note, the CuVOx electrode shows even decreased activity than the Cu electrode, which demonstrate the interaction of Cu and VOx is not the reason for the improved HER activity. In addition, as suggested by the reviewer, the O1s XPS data in Fig. S11 was re-fitted and it is updated into Supplementary Fig. S14 in the Supplementary Information.

**Fig. R6.** LSV polarization curves of Cu and CuVOx in 1 M KOH.

Relevant discussion was added into the revised manuscript in “Electronic and atomic structure analysis” and section “3.4. Supplementary HER activity evaluation (Supplementary Fig. S11.)” in Supplementary Information.

(13) There are abundant Ni^2+^ in Ni(Cu)VOx according to XPS (Supplementary Fig. S6a and S11a), while no first-shell of Ni-O is observed in Ni K-edge FT-EXAFS (Fig. 3e). The authors should explain it.

**Response:** Thanks the reviewer’s comments. First, it is important to point out that XPS is a surface technique and XAS used in these experiments is a bulk technique which can result in very different ratio of Ni-O:Ni in data analysis of the same material.

In the EXAFS fits of both Ni(Cu)VOx and NiVOx, NiO contributions were considered. This is given in the Supplementary Fig. S22 in Fits 4 and 5 for NiVOx and Fits 6 and 7 for Ni(Cu)VOx “with” and “without” NiO. A small contribution of NiO is found to improve the fitting to the EXAFS data of NiVOx. The difference between *fcc* Ni and the inclusion of NiO parameters is shown by a comparison of Fit 4 and Fit 5. The improvements in the refinement are present, as indicated by the decrease in the Chi-squared value. The Ni(Cu)VOx was also fit in the same way. In this case, the fit with NiO was close to the fit without NiO. This indicates that the presence or absence of NiO in Ni(Cu)VOx is not above experimental error, but it does not mean it is not there. As can be seen from Fig. 3a in the Ni K-edge, the white line intensity and its position indicate Ni in Ni(Cu)VOx was also mildly oxidized.

Furthermore, a peak associated with NiO is not observed in the Fourier transform of the EXAFS. In EXAFS, one does not expect to see a secondary component like NiO when it is only a small percentage contribution, because in EXAFS, if the sum of the different sinusoidal contributions, many cancel out or superpose. This is shown in Supplementary Fig. S23 for identical paths. This also can cancel non-equivalent paths, making them hard to see when they are only a small component. One can only work out the likely effect of a smaller contribution by fitting the data, which is shown in the Supplementary Information. In this case, as the structure of metallic Ni is known, the fit is well defined by restraining these parameters in the EXAFS fit.

Relevant discussions have been added into the revised manuscript in “Electronic and atomic structure analysis” and Supplementary Fig. S22 in Supplementary Information.

(14) Although both V-O first-shells were observed for NiVOx and Ni(Cu)VOx in Supplementary Fig. S17, the bridge of “Ni-O-VOx” and the interaction between Ni and V have not been clearly verified. Because the first-shell of V-O in pure VOx may be similar with the V K-edge FT-EXAFS of NiVOx and Ni(Cu)VOx. Thus, the V K-edge XANES and FT-EXAFS of pure VOx should be provided for comparison. Besides, if there exists an O-bridged “Ni-O-VOx”, the second scatting of V-M (V/Ni/Cu) could be observed in V K-edge FT-EXAFS. The unusual second shell in Supplementary Fig. S17 should be explained. It is very crucial to exclude mixed isolated species VOx in the catalysts.

**Response:** We thank the reviewer for the comments. In the original manuscript XAS data, we did not interpret the presence of the bridged Ni-O-VOx unambiguously from the EXAFS. We interpreted that information from the XANES, as the EXAFS was not of a sufficient resolution for this. Please note that there are experimental limitations when analyzing these samples, which unfortunately limit the EXAFS signal to noise ratio. In our sample, VOx is only a minor constituent of the Ni(Cu) matrix. The energy of this XAS data is also low which limits our ability to get high quality EXAFS. In practical terms, we can only get low k-range data, to k = 10.5, which is fine to rule out V-Ni bonding.

We interpreted the presence of the Ni-O-VOx bridges species from the energy position of the pre-edge feature. In Fig. 3d in the manuscript, the positions changes are consistent with a change in Z_eff_. These are well above experiment resolution and provide unambiguous evidence for the presence of Ni-O-VOx interaction. We can fit the second sphere into the EXAFS, as we do in Supplementary Fig. S22. However, it would be over-interpreting to say this is the only way this data can be fit.

In the revised Supplementary Information, we have re-written the text describing the V-EXAFS fits to make this clearer. As V forms only a small amount of the material, it is a very sensitive probe of its local environment. EXAFS taken at the V K-edge of Ni(Cu)VOx and NiVOx were both very weak, compared to the metallic materials. This is clear from the lower intensity of the Fourier transform data. Weak EXAFS is often associated with disordered materials. In this case, the likely explanation is that the VOx takes on a number of slightly different but related geometries. Both the EXAFS of Ni(Cu)VOx and NiVOx were well fit with split inner sphere V-O distances at experimentally reasonable distances of 1.65/1.81 Å, but the noise level of the data would give these a ± 0.15 Å error. Note that for both Ni(Cu)VOx and NiVOx samples, a split inner sphere was necessary to fit the data. Attempts were made to fit a direct Ni-V bond at around 2-2.1 Å. This would be consistent with a scenario where the XANES changes were related to an alloy. However, the data could not be fit this way. The most plausible explanation for the observations at the EXAFS and that also explains the XANES is that the VOx is likely bridged to the Ni *via* an oxygen group, i.e. a Ni-O-VOx interaction. Such an interaction explains both the split coordination sphere, the short V-O bonds and the observations at the XANES.

Additionally, following the suggestion of the reviewer, we further took the XAS data for VOx material deposited under the same conditions. As seen from Fig. R7a, b, the direct interaction between Ni and VOx is clearly seen. In the XAS pre-edge, the effective nuclear charge of the Ni(Cu)VOx and NiVOx is observed at lower energy relative to VOx. In addition, we have also collected the XPS data for the pure VOx, Ni(Cu)VOx and NiVOx. As seen from Fig. R7c, compared with Ni(Cu)VOx and NiVOx, the characteristic peak of pure VOx sample located at a higher binding energy position, which not only indicates it has a higher valence state, but further means there is a direct interaction between Ni and VOx. In terms of the second shell for Ni(Cu)VOx in V K-edge FT-EXAFS, this may be caused by the surface adsorbed VOx. However, the different position of it in comparison with VOx and V_2_O_5_ further indicates the Ni-O-VOx interaction (Fig. R7b).

Relevant discussion was added into the revised manuscript in “Electronic and atomic structure analysis” and Supplementary Fig. S21 and Fig. S25 in Supplementary Information.

**Fig. R7.** (a) V K-edge XANES and (b) its corresponding FT-EXAFS. (c) V2p XPS spectra of VOx, NiVOx and Ni(Cu)VOx.

(15) The deviation in the fitting results of FT-EXAFS should be provided for the judgment of fitting degree.

**Response:** Thanks the reviewer’s comments. Understanding the degree of error in the Fourier transform is complex, one way it is evaluated as part of the fitting is by inspection looking at the algorithmic dependence of the Fourier transform calculations, to make sure noise is not interpreted. A simple way to estimate this noise level in any Fourier transform is to look at the high radial distance to measure the inherent background noise. Also, errors on fitting refinements are given in the Supplementary Information Table S1. In terms of V K-edge, these give an error of ± 0.15 Å on the inner sphere distances.

Relevant discussion is added into section “3.8. Supplementary expanded details of the EXAFS fits” in Supplementary Information.

(16) The authors think that the Cu substitutes the Ni sites and the theoretical models are built in this way. Therefore, the Cu K-edge FT-EXAFS for Ni(Cu)VOx should be provided, together with the Ni K-edge FT-EXAFS.

**Response:** Thanks the reviewer’s comments. At the time of submission, we had not been able to collect Cu EXAFS on these materials. Fortunately, we have a chance to re-take the XAS beamline. We have updated Fig. 3b in the manuscript to include the EXAFS and also present the Cu K-edge FT-EXAFS. It is clear from the Fig. R8, by comparing between the XAS data, the similar XAS feature at Cu K-edge indicates Cu do indeed substitution the Ni site in Ni(Cu)VOx.

We have added this discussion into “Electronic and atomic structure analysis” in the revised manuscript and Supplementary Fig. S25b in Supplementary Information.

**Fig. R8.** (a) Cu K-edge XAS of the prepared samples and (b) its corresponding FT-EXAFS.

(17) In situ XAS and Raman spectroscopy could provide more valuable information for the active sites of the catalysts.

**Response:** Thanks for the reviewer’s comments to improve our manuscript. We completely agree that in situ XAS and Raman would be ideal to provide information on the active phases of the catalysts. However, due to the limited beamline source and special requirements for in situ XAS, we have been unable to carry out in situ XAS experiments. Instead, we have tried to perform the in-situ Raman spectroscopy at an applied negative potential of –1.2 V (*vs*. Ag) under HER condition by mounting the Ni(Cu)VOx electrode onto a screen-printed electrode. However, the generated hydrogen bubbles severely affected the laser beam and thus no signal can be collected. Therefore, we applied a semi in-situ approach to measure our sample. That is, the Raman spectra were collected immediately after 20 min HER reaction. As seen from Fig. R9, compared with the Ni(Cu) electrode, the shift of the Raman spectra for Ni-O bind indicates a direct interaction between VOx and Ni. For the Ni(Cu)VOx Raman spectra collected at open circuit potential (OCP) and at –1.2 V (*vs*. Ag), there is a clear decrease on peak intensity for V-O at 800-900 cm^-1^, which indicates the dissolution of the physically absorbed and weakly bonded VOx. In addition, there is also a slight peak decrease for Ni-O, which can be attributed to the reduction of Ni-O at negative potential. However, the interaction between VOx and Ni still existed, which is responsible for the enhanced HER activity, as evidenced from ex situ XAS and DFT calculations.

The relevant discussion is added into the revised manuscript in “Discussion” section and Supplementary Fig. S33 in the Supplementary Information.

**Fig. R9.** Raman spectroscopy for Ni(Cu)VOx collected at OCP and semi in-situ condition after applying a –1.2 V (*vs*. Ag) for 20 min and the Ni(Cu) electrode under OCP.

(18) The description about VOx species in lines 252-265 may misguide readers to think that the V ions occupy the Ni sites in the lattices.

**Response:** We have updated the description accordingly as per response to question # 8.

(19) The specified exposed area of NF electrode for electrochemical measure should be provided.

**Response:** The exposed surface area of the NF electrode is 0.5 cm × 0.5 cm = 0.25 cm^2^. This information is added into the revised manuscript in “Electrochemical measurement” in “Methods” section.

**Response to Reviewer: #3**

This paper reports on tuned nickel-based catalysts as electrocatalysts for hydrogen evolution reaction (HER) for alkaline water electrolysis. Ni, Cu and VOx are formed on the surface of nickel foam by electrodeposition method, and VOx plays an important role in excellent HER activity. The authors also suggest the causes of activity and durability through various characterizations and DFT calculations.

(1) Ni (Cu) VOx was formed on two supports, Nickel foam (NF) and FTO. The authors explain the use of the FTO for physical characterization purposes. This approach is understandable, but the reviewer believes that it is necessary to confirm that the active metals loaded on two different supports are in the same state. Several methods may be considered, such as XPS (Depth profiling) or ICP analysis of surface scraped powder.

**Response:** Thanks very much for the reviewer’s comments. As suggested by the reviewer, we tested the Ni(Cu)VOx powder scraped from nickel foam by ICP-OES analysis. The doped Cu and V were determined to be Cu : Ni = 0.12 and V : Ni = 0.034 (molar ratio), respectively. This is close to the Cu : Ni = 0.114 and V : Ni = 0.029 (molar ratio) that measured by electrodeposition of the sample on FTO.

This information is added into the revised manuscript in “Evaluation of electrocatalytic activity and stability” section.

(2) Considering the standard reduction potential of Ni, Cu, V or VOx, at -2.0 V (vs. SCE), all elements are expected to be electrodeposited simultaneously. In this case, the composition of each layer (by thickness) may be different due to the difference in the reduction properties of each metal. However, in this paper, the analysis of the structure of the active metal is not clear. It is also likely that at significantly lower potentials, such as -2.0 V, vanadium precursors may be reduced to V (s) as well as VOx. The authors confirm all existing forms as VOx and continue discussion.

**Response:** Thanks the reviewer’s comments. It is true that at –2.0 V (*vs*. SCE), the precursors of Ni, Cu and V all can be electrodeposited simultaneously onto the nickel foam substrate, as can be seen from Fig. R10. The different deposition potential of each metal can influence the electrodeposition process and subsequently the composition of the film. However, in our case, the Ni^2+^ concentration (0.5 M) is 40 times higher than Cu^2+^ and 71.4 times higher than VO^3-^, therefore Ni deposition dominate, especially at the very negative potentials e.g. –2.0 V. This is evident that at negative potentials (< –1.25 V, highlighted in Fig. R10), the deposition curve is very close to that of Ni, indicating Ni is the dominate element in the electrodeposited film, whilst Cu and V are doped into the structure of Ni. We also have carried out the XPS depth profile, and it shows Cu and V are uniformly distributed throughout the Ni(Cu)VOx composite (see response to question #4). These results are also supported by the XRD and ICP-OES data, as we discussed in the manuscript.

Furthermore, to see if the ammonium metavanadate precursor can be reduced to metallic V, we obtained the XRD spectra of the electrodeposited pure VOx on both FTO and carbon fiber paper. As seen from Supplementary Fig. S5, there is no new peaks appeared that can be assigned to metallic V. This result is in consistence with the XPS and XAS data, where only oxidized V is detected. In addition, previous reports show that metallic V can be obtained *via* electro-reduction of sodium metavanadate in molten salt at high temperatures (> 1,000 K, *Int. Journal of Refractory Metals and Hard Materials*, **55**, 47-53 (2016)), while room temperature electrochemical reduction can only achieve oxidized vanadium state (*Electrochimica Acta*, **76**, 262-269 (2012)).

Relevant discussion is added into the revised manuscript in “Synthesis and characterization of the Ni(Cu)VOx catalyst” and Supplementary Fig. S3 and Fig. S5 in Supplementary Information.

**Fig. R10.** The cyclic voltammetry curves of electrochemical reduction behaviors of Ni^2+^, Cu^2+^ and VO^3-^.

(3) Further explanation is needed for the expressions Cu^2+^/Ni^2+^ = 2.5% and Cu/Ni = 11.4%. The ratio expression and the "%" unit are used together, so the meaning is not clear. In order to compare HER electrochemical activity, it is important to know the Cu/Ni and V/ Ni ratio information together with the absolute amount of active metal loaded in NF. Although the text says "Catalyst mass loading on NF is: (3.0 +/- 0.2) mg/cm^2^", it is thought that the ratio of each metal and the "absolute amount of metal" should be provided together.

**Response:** Thanks the reviewer’s comments. We have changed the expression into “the best Ni(Cu)VOx HER electrode was prepared by electrodeposition at –2.0 V (*vs*. SCE) in an electrolyte bath containing 0.5 M NiSO_4_, 12.5 mM CuSO_4_, 7 mM NH_4_VO_3_ and 0.5 M H_3_BO_3_” and “The doped Cu and V were determined to be Cu : Ni = 0.114 and V : Ni = 0.029 (molar ratio) by using inductively coupled plasma optical emission spectroscopy (ICP-OES)”. Thus, the absolute amount of each metal is calculated to be ~2.6 mg for Ni, ~0.3 mg for Cu and ~ 0.1 mg for V in the composite.

This information is added into the revised manuscript in “Evaluation of electrocatalytic activity and stability” and “Methods” section.

(4) After electrodeposition for 600 seconds, galvanic displacement reactions of “vanadium and/or nickel metals” and Cu can occur if Cu ions remain in the precursor solution and are contained in the electrolyte for a short time under OCV conditions. In this case, the surface of the active metal is probably rich in Cu. These experimental variables can make a real difference in the structure of the metal and lead to different results than the basic structure used in the DFT. In spite of the results of Fig. 3 (a), the authors do not consider the possibility of existing nickel in NiOx form in the DFT.

**Response:** Thanks the reviewer’s comments. In our case, to prevent the displacement reactions happens; all the prepared electrodes were removed immediately from the electrolyte after deposition and washed thoroughly with Milli-Q water. We don’t find in our experiments the surface is rich in Cu, which can be evidenced from the XPS depth profile (Fig. R11). In the XPS depth profile, both the Ni and Cu amount was increased with etching time, along with the decrease of O. Then, the amount of the each element is stabilized, which indicates Cu and V are uniformly distributed throughout the Ni(Cu)VOx composite.

In order to consider the possibility of existing nickel in NiOx form on catalyst surface, we constructed a DFT model with one layer NiO on Ni(111) surface (NiO/Ni(111)). The calculated ${\Delta G}_{H^{*}}$ of H^*^ adsorbed on NiO/Ni(111) is –0.38 eV (Fig. R12), which is very close to that on bare Ni(111) (–0.32 eV), indicating that the HER activities of NiO/Ni(111) and Ni(111) are similar.

We have made relevant discussion in the revised manuscript in “Theoretical simulations” and Supplementary Fig. S28 and Fig. S31 in Supplementary Information.

**Fig. R11.** XPS depth profile of Ni(Cu)VOx.

**Fig. R12.** Top (upper) and side (lower) views of the optimized structures of (a) bare NiO/Ni(111) and (b) H^*^ adsorbed on NiO/Ni(111). The calculated ${\Delta G}_{H^{*}}$ of H^*^ adsorbed on NiO/Ni(111) are listed in (b).

(5) The authors argue that VOx plays a major role in HER response activity and durability. In 'parameter fitting' and 'structure for DFT calculation', the position and role of VOx are not clear. In terms of durability, only the dissolution of "physically absorbed and weakly bonded VOx" is mentioned. Even after AST, large amounts of V are still observed. Further analysis of the location and role of V (s) or VOx seems necessary.

**Response:** Thanks the reviewer’s comments. From Fig. 1e-h the TEM EDS mapping in the manuscript and the XPS depth profiling (Fig. R11 our response to previous question), VOx are distributed uniformly in the composite. Thus, in the revised version, we constructed a DFT model of Ni(111) doped with VO_3_ cluster inside Ni lattice (Fig. R13) to simulate the case that VOx located in Ni lattice. We find that the ${\Delta G}_{H^{*}}$ at site 1 is –0.24 eV, which is less negative than that on bare Ni(111) (–0.32 eV). This indicates that VOx located inside Ni lattice can also enhance the HER activity. However, compared to the case that VOx located on Ni(111) surface, the HER enhancement effect is small. Please note that ${\Delta G}_{H^{*}}$ at other sites for Ni(111) with VO_3_ cluster inside Ni lattice are comparable or more negative (–0.32 ~ –1.16 eV) than that on bare Ni(111) (–0.32 eV).

In terms of the roles of VOx, the deformation electronic density calculation (Fig. 4e-f in the manuscript) and Bader analysis show that the VOx cluster has led to an increase in electronic charge density on VOx and a loss of electron charge density on the surrounding three Ni atoms, and there is 0.22 electron transferred from each Ni to VOx based on the Bader analysis. Less electrons localized on Ni sites closest to VOx results in a closer optimal value of $\left| {\Delta G}_{H^{*}} \right|$ on Ni(111), and the enhanced HER activity.

In regard to the durability, we attribute the decrease of activity to the partial dissolution of physically absorbed or weakly bonded VOx with Ni in the composite that dissolved in alkaline electrolyte. This is because the concentrations of Ni and Cu in the electrolyte remain stable at 0.08 mg mL^-1^ and 0.04 mg mL^-1^ after stability test, indicating there is no dissolution or peeling off during the long-term stability test.

Relevant discussion is highlighted in the revised manuscript in “Theoretical simulations”, “Evaluation of electrocatalytic activity and stability” sections and Supplementary Fig. S32 in Supplementary Information.

**Fig. R13.** (a) The optimized structure of VO_3_ cluster in Ni lattice. (b) The optimized structure of H* adsorbed at site 1 on Ni(111) surface with VO_3_ cluster in Ni lattice. The black numbers in (a) denote the possible adsorption sites for H* closed to the oxidized V cluster VO_3_, and the corresponding ${\Delta G}_{H^{*}}$ are listed in the table on the bottom.

(6) Information about the Electrolyte used in the Electrodeposition process needs to be added.

**Response:** Thanks the reviewer’s comments. We have put the details of the electrolyte in the revised manuscript in “Methods” section and Supplementary Information. Take the Ni(Cu)VOx as an example, the electrodeposition was conducted in an electrolyte containing 0.5 M NiSO_4_, 12.5 mM CuSO_4_, 7 mM NH_4_VO_3_ and 0.5 M H_3_BO_3_, by dissolving each chemical in 50 mL Milli-Q water under sonication. The electrolyte to deposit other control samples can be found in the Supplementary Information.

(7) ECSA calculation results can be correlated with BET specific surface area results.

**Response:** Thanks the reviewer’s suggestion. The measured BET specific surface area for Ni, Ni(Cu), NiVOx and Ni(Cu)VOx are 17.988 m^2^ g^-1^, 26.614 m^2^ g^-1^, 18.994 m^2^ g^-1^ and 34.232 m^2^ g^-1^, respectively (Fig. R14). As can be seen, the Ni(Cu)VOx still shows the largest specific surface area. Thus, the two techniques show the same trend in surface area. A difference in the measured value between these two techniques is expected due to different working mechanisms, where BET area measures the physiosorption of N_2_ through the Van der Waals force in gas phase, while ECSA measures double-layer capacitance between the electrode and electrolyte.

Relevant discussion is added into the revised manuscript in “Electronic and atomic structure analysis” section and Supplementary Fig. S20 in Supplementary Information.

**Fig. R14.** N_2_ adsorption/desorption isotherms of the prepared catalysts.

(8) It would be nice to express that Fig. 2 (e) is vanadium. In addition, Ni and Cu information as well as vanadium may be helpful in comparing durability.

**Response:** Thanks the reviewer’s comments. Yes, we have pointed out Fig. 2e is for vanadium. Furthermore, the concentrations of Ni and Cu in the electrolyte after stability with 0.08 mg mL^-1^ and 0.04 mg mL^-1^, respectively, also have been added into the revised manuscript, which indicates there is no dissolution or peeling off the Ni and Cu from the catalysts during the long-term stability test.

This information together with stability discussion has been added in the “Evaluation of electrocatalytic activity and stability” section.

(9) It appears that "Ar gas" and "N_2_ flow rate" are used simultaneously in "3.6 Supplementary".

**Response:** Thanks the reviewer. This has been revised in section “3.6. Supplementary Faradaic efficiency testing” in the Supplementary Information.

In summary,

It is clear that the Ni-based Non-PGM catalyst presented in this paper shows very good HER activity. The authors also attempted to suggest the structure of the catalyst and the reason for its high activity through XAS analysis and DFT. However, there is still some ambiguity in the structure and discussion. After some key updates, the reviewer suggests that this article may be considered for publication in this or other journals.

**Response:** We appreciate the reviewer’s comments for improving our work, and hope the above responses have addressed the reviewer’s concerns and the revised manuscript is acceptable for publication

**Reviewer comments, second version:**

REVIEWERS' COMMENTS:

Reviewer #1 (Remarks to the Author):

The authors have carefully addressed the comments of the reviewers and the manuscript is now suitable for publication.


Reviewer #2 (Remarks to the Author):


All my concerns have been well addressed or explained in the revised manuscript and authors' responses. In my opinion, the revised manuscript could be published in Nat. Commun.


Reviewer #3 (Remarks to the Author):

This is a 2n round review process. The authors have tried to resolve the comments from the reviewer, and the materials presented are believed to have novelty.
One minor update is required. In the expression for metal composition, it is considered that the form of “A: B = X: Y” or “A / B = X” is a clear expression. Current status like "A: B = 0.12" needs updating.

**Author rebuttal, second version:**

**Reviewer #1 (Remarks to the Author):**

The authors have carefully addressed the comments of the reviewers and the manuscript is now suitable for publication.

**Response:** We appreciate the reviewer’s suggestion of acceptance of our work for publication in *Nature Communications.*

**Reviewer #2 (Remarks to the Author):**

All my concerns have been well addressed or explained in the revised manuscript and authors' responses. In my opinion, the revised manuscript could be published in Nat. Commun.

**Response:** We appreciate the reviewer’s suggestion of acceptance of our work for publication in *Nature Communications.*

**Reviewer #3 (Remarks to the Author):**

This is a 2n round review process. The authors have tried to resolve the comments from the reviewer, and the materials presented are believed to have novelty. One minor update is required. In the expression for metal composition, it is considered that the form of “A: B = X: Y” or “A / B = X” is a clear expression. Current status like "A: B = 0.12" needs updating.

**Response:** Thanks for the reviewer’s suggestion. We have updated the expression for metal composition into A/B = X in the revised manuscript.

**Reviewer comments, third version:**

**Author rebuttal, third version:**
